# Biophysical basis for Kv1.3 regulation of membrane potential changes induced by P2X4‐mediated calcium entry in microglia

**DOI:** 10.1002/glia.23847

**Published:** 2020-06-11

**Authors:** Hai M. Nguyen, Jacopo di Lucente, Yi‐Je Chen, Yanjun Cui, Rania H. Ibrahim, Michael W. Pennington, Lee‐Way Jin, Izumi Maezawa, Heike Wulff

**Affiliations:** ^1^ Department of Pharmacology University of California Davis California USA; ^2^ Department of Pathology and Laboratory Medicine and M.I.N.D. Institute University of California Davis Medical Center Sacramento California USA; ^3^ AmbioPharm Inc. North Augusta South Carolina USA

**Keywords:** intracellular Ca^2+^, Kir2.1, Kv1.3, membrane potential, microglia, P2X4, P2X7, PAP‐1, potassium channels, purinergic receptor, ShK‐223

## Abstract

Microglia‐mediated inflammation exerts adverse effects in ischemic stroke and in neurodegenerative disorders such as Alzheimer's disease (AD). Expression of the voltage‐gated potassium channel Kv1.3 is required for microglia activation. Both genetic deletion and pharmacological inhibition of Kv1.3 are effective in reducing microglia activation and the associated inflammatory responses, as well as in improving neurological outcomes in animal models of AD and ischemic stroke. Here we sought to elucidate the molecular mechanisms underlying the therapeutic effects of Kv1.3 inhibition, which remain incompletely understood. Using a combination of whole‐cell voltage‐clamp electrophysiology and quantitative PCR (qPCR), we first characterized a stimulus‐dependent differential expression pattern for Kv1.3 and P2X4, a major ATP‐gated cationic channel, both in vitro and in vivo. We then demonstrated by whole‐cell current‐clamp experiments that Kv1.3 channels contribute not only to setting the resting membrane potential but also play an important role in counteracting excessive membrane potential changes evoked by depolarizing current injections. Similarly, the presence of Kv1.3 channels renders microglia more resistant to depolarization produced by ATP‐mediated P2X4 receptor activation. Inhibiting Kv1.3 channels with ShK‐223 completely nullified the ability of Kv1.3 to normalize membrane potential changes, resulting in excessive depolarization and reduced calcium transients through P2X4 receptors. Our report thus links Kv1.3 function to P2X4 receptor‐mediated signaling as one of the underlying mechanisms by which Kv1.3 blockade reduces microglia‐mediated inflammation. While we could confirm previously reported differences between males and females in microglial P2X4 expression, microglial Kv1.3 expression exhibited no gender differences in vitro or in vivo.

**Main Points:**

- The voltage‐gated K^+^ channel Kv1.3 regulates microglial membrane potential.
- Inhibition of Kv1.3 depolarizes microglia and reduces calcium entry mediated by P2X4 receptors by dissipating the electrochemical driving force for calcium.

## Main Points

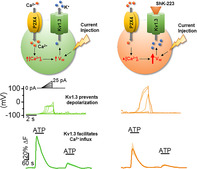

## 1 INTRODUCTION

Microglia constitute the main immunocompetent cells of the central nervous system (CNS) and play a major role in the maturation of neuronal networks in the developing brain and in the maintenance of homeostasis in adults (Kettenmann, Hanisch, Noda, & Verkhratsky, 2011). In vivo microglia exist in a surveillant state and are constantly scanning their surroundings with highly mobile processes for damaging or pathological signals (Nimmerjahn, Kirchhoff, & Helmchen, 2005). Depending on the nature of the recognized pathogen‐associated molecular pattern (PAMP) or damage‐associated molecular pattern (DAMP) signal, microglia can quickly become activated to resolve or mediate exaggerated neuroinflammation as seen in many CNS‐associated pathologies such as ischemic stroke and Alzheimer's disease (AD) (Perry & Teeling, 2013; Ransohoff, Schafer, Vincent, Blachere, & Bar‐Or, 2015).

Activated microglia are often classified according to their peripheral macrophage counterparts as either “classically” or “M1” activated and “alternatively” or “M2” activated when stimulated with interferon‐γ or interleukin‐4 (IL‐4), respectively. While this classification is now widely viewed as overly simplistic and advocated against based on new knowledge from omic technologies (Ransohoff, 2016), it remains a useful concept for mechanistic and therapeutic hypothesis generation for associating the M1‐state with a pro‐inflammatory and neurotoxic role of microglia, and the M2‐state with a reparative and neuroprotective role. Our recent in vitro studies showed that the expression of the voltage‐gated potassium channel Kv1.3 is preferentially increased in lipopolysaccharides (LPS)‐differentiated M1‐like but not IL‐4‐differentiated M2‐like microglia (Nguyen et al., 2017). Similarly, in vivo Kv1.3 expression is upregulated in microglia from intracerebroventricular (ICV)‐LPS injected brains (Di Lucente, Nguyen, Wulff, Jin, & Maezawa, 2018). Elevated Kv1.3 expression is also observed in activated microglia from rodents and humans with AD (Maezawa et al., 2018; Rangaraju, Gearing, Jin, & Levey, 2015) and following ischemic stroke (Chen et al., 2016; Chen, Nguyen, Maezawa, Jin, & Wulff, 2018), making Kv1.3 an attractive target for immunomodulatory therapy. Accordingly, our group showed that both genetic deletion and pharmacological blockade of Kv1.3 diminished microglial activation and concomitant inflammatory responses, leading to improved pathological and neurological outcomes in multiple animal models of neuroinflammation (Chen et al., 2018; Di Lucente et al., 2018; Maezawa et al., 2018).

Despite these positive outcomes, the mechanisms underlying the effectiveness of Kv1.3 inhibition in alleviating pro‐inflammatory microglia functions is currently unclear and has only been inferred from results obtained with T‐cells but not examined experimentally in microglia. In T‐cells, where Kv1.3 has been studied extensively and deemed essential for maintaining a negative membrane potential, blocking Kv1.3 disrupts calcium signaling mediated by the calcium release‐activated channels (CRAC) during T‐cell activation (Cahalan & Chandy, 2009; Leonard, Garcia, Slaughter, & Reuben, 1992; Lewis & Cahalan, 1995) and downstream functions such as NFAT translocation, IL‐2 release, and proliferation (Beeton et al., 2006; Negulescu, Shastri, & Cahalan, 1994). The goal of this study, therefore, was to investigate the contribution of Kv1.3 channels to microglial membrane potential and calcium signaling. Specifically, we investigated Kv1.3's involvement in the regulation of microglial Ca^2+^ signaling facilitated by the P2X4 and P2X7 receptors since similar to Kv1.3, these purinergic receptors have been associated with inflammatory microglia functions in ischemia or following LPS activation (Kettenmann et al., 2011).

Here, we first used the current‐clamp technique to probe the fundamental significance of Kv1.3 in maintaining membrane potential at rest and in limiting evoked depolarization changes in Chinese Hamster Ovary (CHO) cells and microglia. We then examined the role of Kv1.3 in handling microglial membrane depolarizations caused by the rise in intracellular Ca^2+^ following activation of P2X4 with ATP. We found that Kv1.3 channels contribute to the regulation of microglia membrane potential both at rest and during active calcium signaling. The selective peptide inhibitor ShK‐223 depolarized microglial membrane potential, disabled the ability of Kv1.3 to clamp ATP‐induced depolarization (AID) and effectively reduced calcium entry via the P2X4 receptors. Taken together, our data suggest that Kv1.3 blockers exert their immunomodulatory effects by disrupting the channel's ability to maintain a negative driving force for Ca^2+^ entry at rest and to buffer excessive depolarizations during active calcium signaling.

## 2 MATERIALS AND METHODS

### 2.1 Cells and constructs

CHO cells were obtained from the American Type Culture Collection (ATTC, Manassas, VA) and were maintained in Dulbecco's modified Eagle's medium (DMEM; Gibco, Carlsbad, CA) supplemented with 10% fetal bovine serum (FBS) and 1% penicillin/streptomycin in a humidified 5% CO_2_ incubator at 37°C. Cells were transfected using the FuGene 6 Transfection Reagent (Promega) following the manufacturer's instructions and were used for electrophysiology 24–48 hr after transfection. Human Kv1.3 cDNA (Gene Bank accession number L23499) cloned in the pRC‐CMV vector was a generous gift from Dr Carol Deutsch (University of Pennsylvania, Philadelphia, PA). The rat P2X4 construct (Gene Bank accession number X93565) cloned into the pCDNA3 vector was kindly provided by Dr. Kenton Swartz (National Institute of Neurological Disorders and Stroke). Each clone was co‐transfected with pEGFP‐C1 cDNA (Invitrogen) at a 3:1 ratio of channel to pEGFP for identification of transfected cells under an epifluorescence microscope for electrophysiology.

### 2.2 Animals

Timed pregnant female C57BL/6J mice were purchased from Charles River Laboratories. Male homozygous CX_3_CR1^GFP/GFP^ knock‐in mice (Jung et al., 2000) were obtained from The Jackson Laboratory as B6.Cg‐Ptprc^a^ CX3CR1tm1Litt/LittJ (strain 005582) and crossed with female C57BL/6J mice by the Mouse Biology Program of the University of California, Davis to obtain CX_3_CR1^+/GFP^ heterozygote F1 mice. All protocols involving mouse models were approved by the Institutional Animal Care and Use Committee of the University of California, and were performed in accordance with the U.S. National Research Council's Guide for the Care and Use of Laboratory Animals, the U.S. Public Health Service's Policy on Humane Care and Use of Laboratory Animals, and the Guide for the Care and Use of Laboratory Animals. The surgical procedures (ICV‐LPS injection and middle cerebral artery occlusion [MCAO]) were performed in accordance with the guidelines for survival surgery in rodents of the University of California.

### 2.3 Primary cultures of mouse microglia

Primary microglia were prepared from postnatal P0‐P1 C57BL/6J mice as described previously (Maezawa, Zimin, Wulff, & Jin, 2011) but with minor modifications to culture cells into separate male, female, or mixed‐gender cultures. After 7–14 days in culture, floating microglia were harvested with the “shaking off” method and reseeded at 100,000–200,000 cells per well in 24‐well plates in DMEM (25 mM glucose) supplemented with 10% FBS, 1 mM Na^+^ pyruvate, 100 units/ml penicillin, and 100 μg/ml streptomycin. After 2–4 hr, DMEM with 10% FBS was replaced with DMEM containing 2% FBS and cells were cultured overnight before differentiated into either M1 or M2 phenotypes by a 24‐hr stimulation with 300 ng/ml LPS (*Escherichia coli* 0111:B4, Millipore Sigma) or 20 ng/ml IL‐4 (Millipore Sigma), respectively, in reduced serum growth media with 2% FBS.

### 2.4 ICV injection of LPS

LPS (*E. coli* O55:B5, Millipore) dissolved in phosphate‐buffered saline (PBS) or PBS only as a vehicle was ICV administered stereotactically to 12‐week‐old male C57Bl/6J mice as previously described (Di Lucente et al., 2018). Briefly, a total volume of 2 μl was injected per side into the lateral ventricles of anesthetized mice using a Hamilton syringe with a 27‐gauge needle (Hamilton, Reno, NV). Injection sites were coordinated at −1 mm posterior to the bregma, 1.3 mm lateral to the sagittal suture, and 2 mm in depth. The incision was surgically closed and mice were placed on an isothermal pad at 36°C and continuously monitored following surgery until recovery. Twenty‐four hours later, mice were euthanized, and the brain tissues were processed for isolation of CD11b + microglia for quantitative PCR (qPCR) analysis.

### 2.5 Middle cerebral artery occlusion

Microglial Kv1.3, Kir2.1 and P2X4 expression was studied in brains of 16 week‐old male or female CX_3_CR1^+/GFP^ mice subjected to transient MCAO surgery (60 min occlusion) with 8 days of reperfusion as described previously (Chen et al., 2018).

### 2.6 Acute microglia isolation

Microglia isolation using Miltenyi CD11b‐conjugated magnetic microbeads was described previously (Chen et al., 2018). Briefly, brains were quickly dissociated enzymatically with a Neural Tissue Dissociation Kit (Miltenyi Biotec). Microglia were subsequently purified by magnetic‐activated cell sorting (MACS) using anti‐CD11b magnetic beads (Miltenyi Biotec). The whole process took about 60–90 min and isolated cells were immediately used for downstream applications without culturing or exposure to serum.

### 2.7 Electrophysiology

Recordings from cultured and ex vivo acutely isolated microglia were performed on cells that were plated on poly‐lysine‐coated glass coverslips immediately after trypsin–EDTA detaching or isolation, respectively. All electrophysiological measurements started after cells were incubated at 37°C for 10 min. Currents were recorded using the whole‐cell configuration of the patch‐clamp technique at room temperature with an EPC‐10 HEKA amplifier and the HEKA PatchMaster 9 data acquisition software. External normal Ringer solution contained 160 mM NaCl, 4.5 mM KCl, 2 mM CaCl_2_, 1 mM MgCl_2_, 10 mM HEPES, pH 7.4, and 300 mOsm. Patch pipettes were pulled from soda lime glass (micro‐hematocrit tubes, Kimble Chase, Rochester, NY) to resistances of 2–3 MΩ when submerged in the bath solution and filled with an internal solution containing 160 mM KCl, 2 mM MgCl_2_, 10 mM HEPES, and 10 mM EGTA, pH 7.2, 300 mOsm. Kv1.3, Kir, P2X4, and P2X7 currents were recorded in the voltage‐clamp mode. K^+^ currents were elicited with 200‐ms voltage ramps from −120 to +40 mV at a frequency of 0.1 Hz. Inward rectifier (Kir) currents were measured as peak inward currents at −120 mV. Kv1.3 currents were measured as PAP‐1‐ or ShK‐223‐sensitive delayed rectifier outward currents elicited at voltages positive to −40 mV from the same cell. Kir and Kv1.3 current densities were determined by dividing their current amplitudes in picoamperes (pA) at −120 mV (Kir) or + 40 mV (Kv1.3) by the cell capacitance measured in picofarads (pF). P2X currents were elicited by an application of either a 2‐s pulse of ATP or a 3‐s pulse of BzATP using triple‐barreled theta glass and a Perfusion Fast‐Step SF‐77 System (Warner Instruments, Hamden, CT) at a holding potential of −60 mV. P2X current densities were determined by dividing their current amplitudes in picoamperes (pA) by the cell capacitance measured in picofarads (pF). Cell capacitance was directly measured by a lock‐in function built in the HEKA PatchMaster 9 data acquisition software. Resting membrane potential (RMP) and membrane depolarization induced by current ramp steps or ATP were measured in current‐clamp mode. Seal and access resistance, as well as cell capacitance, a direct measurement of cell surface area, were monitored continuously throughout all recordings. Whole‐cell patch‐clamp data are presented as mean ± *SD*. Data were analyzed using one‐way analysis of variance (ANOVA) followed by post hoc Tukey–Cramer's method to determine statistical significance in every figure where three groups or more were compared. Measurements made within the same group of cells before and after treatment with ShK‐223 or ivermectin were compared using a paired *t* test. All comparisons made between two groups of cells subjected to different stimulation condition (e.g., untreated vs. LPS stimulated) are analyzed by unpaired *t* test. Pearson *r* correlation was calculated using Origin 9.0 (OriginLab, Northampton, MA) with significant correlation determined at *p* < 0.05.

### 2.8 Reagents

The selective Kv1.3 small molecule inhibitor PAP‐1 was synthesized in our laboratory as previously described (Schmitz et al., 2005). ShK‐223, a highly Kv1.3‐selective derivative of the sea anemone *Stichodactyla helianthus* toxin peptide ShK (Pennington et al., 2015) was synthesized at AmbioPharm Inc. (North Augusta, SC). Ivermectin, 2′(3′)‐*O*‐(4‐benzoylbenzoyl) adenosine 5′‐triphosphate triethylammonium salt (BzATP), probenecid and adenosine 5′‐triphosphate (ATP) disodium salt hydrate (ATP) were purchased from Millipore Sigma (St. Louis, MO).

### 2.9 Immunofluorescence staining

Before extracting brains from mice 8 days after MCAO, mice were heavily anesthetized and subjected to transcardial perfusion with PBS, followed by perfusion fixation with 4% buffered formalin in PBS. Brains were quickly removed and further immersion‐fixed for an additional 24 hr. After dehydration in 30% sucrose PBS brains were sectioned into four 2‐mm thick coronal slices starting from the frontal pole, embedded in optimal cutting temperature media, and cryosectioned at 14 μm thickness. P2X4 was stained for with a polyclonal rabbit anti‐P2X4 antibody (Alomone, APR‐002, 1:1,000) for 1 hr at RT. Kv1.3 was stained with a polyclonal rabbit anti‐Kv1.3 antibody (Alomone, APC‐101, 1:1,000). Bound primary antibodies were detected by Alexa Fluor®647‐conjugated secondary antibodies (1:1,000, Life Technologies). Slides were mounted in Fluoromount‐G (Southern Biotech) with DAPI and imaged with a Keyence BZ‐X710 fluorescence microscope (Keyence Corporation). Supplementary Figures S2–S4 show that the secondary antibodies do not produce any unspecific staining and that staining by the primary antibodies can be prevented by preincubation with the respective blocking peptides (Alomone).

### 2.10 Quantitative PCR

Total RNA was extracted using RNeasy Plus Mini Kit (Qiagen). For RNA from microglia acutely isolated from adult mouse brain reverse‐transcription and preamplification was performed with Ovation PicoSLWTA System V2 kit (NuGen, San Carlos, CA). For RNA from cultured primary neonatal microglia cDNA was synthesized using iScript Reverse Transcription Supermix (Bio‐Rad, Hercules, CA). RNA purity and concentrations were assessed by measuring the absorbance at 260 and 280 nm using a NanoDrop 2000C Spectrophotometer (Thermo Scientific, Waltham, MA). qPCR was performed using the SsoFast EvaGreen Supermix (Bio‐Rad) in the CFX96 Touch Real‐Time PCR Detection System (Bio‐Rad). The following forward/reverse primer pairs were used:

IL‐1β: 5′‐CCCCAAGCAATACCCAAAGA‐3′/5′‐TACCAGTTGGGGAACTCTG‐3′;

TNF‐α: 5′‐GACGTGGAACTGGCAGAAGAG‐3′/5′‐TGCCACAAGCAGGAATGAGA‐3′;

iNOS: 5′‐CGGATAGGCAGAGATTGGAG‐3′/5′‐GTGGGGTTGTTGCTGAACTT‐3′;

Arg1: 5′‐CCAACTCTTGGGAAGACAGC‐3′/5′‐TATGGTTACCCTCCCGTTGA‐3′;

P2X7: 5′‐TTATGGCACCGTCAAGTGG‐3′/5′‐TCTCCGTCACCTCTGCTATG‐3′;

P2X4: 5′‐GACCAACACTTCTCAGCTTGG‐3′/5′‐GTGACGATCATGTTGGTCATG‐3′.

For Kv1.3 (qMmuCED0049811) and Kir2.1 (qMmuCID0008540), the commercially available primer sets from Bio‐Rad were used. For β‐actin, the commercially available primer set Mouse‐ACTB from Applied Biosystems was used (ThermoFisher catalogue # 4352341E). Relative cDNA levels for the target genes were analyzed by the 2^ΔΔCt method using Actb as the internal control for normalization. For each marker a two‐tailed one‐sample *t* test was performed on the log‐transformed fold‐change value, which amounts to doing a paired test comparing the log‐transformed (unnormalized) values at a given time point to the log‐transformed normalization value for that marker for that replication.

### 2.11 Fluorescence indicator measurement of intracellular Ca^2+^

[Ca^2+^]_i_ measurements were carried out on cultured microglia seeded on glass coverslips at a density of 10,000 cells/100 μl. Real‐time changes of [Ca^2+^]_i_ in microglia were detected using a time‐lapse imaging module on a BZ‐X780 fluorescence microscopy (Keyence LLC, Campbell, CA) using the fluorescent calcium indicator Fluo‐4/AM from Invitrogen (ThermoFisher). To prepare cells for imaging, microglia were rinsed three times with normal Ringers solution and loaded with Fluo‐4/AM (5 μM) for 60 min at room temperature (20–22°C). Subsequently, the coverslips were thoroughly rinsed five times with Ringers solution to remove excess dye and incubated for an additional 30 min to achieve complete deesterification of internalized Fluo‐4/AM. For experiments testing the effect of Kv1.3 inhibition on intracellular calcium changes, cells were preincubated with ShK‐223 (100 nM) during this deesterification step. Coverslips were then mounted on the stage of the microscope and cells were excited at 495 nm and images were captured at 516 nm at 3 s intervals. All fluorescence measurements were made at room temperature from subconfluent areas of the coverslips to ensure that only individual microglia were measured. Throughout the experiment, cells were continuously superfused with electrophysiological Ringers solution containing 2 mM of Ca^2+^ and all solutions were applied through a gravity‐fed perfusion system at a flow rate of 2–3 ml/min. Probenecid (2 mM) was included in all solutions throughout all steps to prevent Fluo‐4 leakage from cells. Baseline fluorescence was recorded for 2 min. Image data were analyzed off‐line and the change in [Ca^2+^]_i_ was represented as ΔF/F (change in fluorescence after baseline subtraction).

## 3 RESULTS

### 3.1 Kv1.3 mediates the resistance to excessive membrane potential changes

**FIGURE 1:**
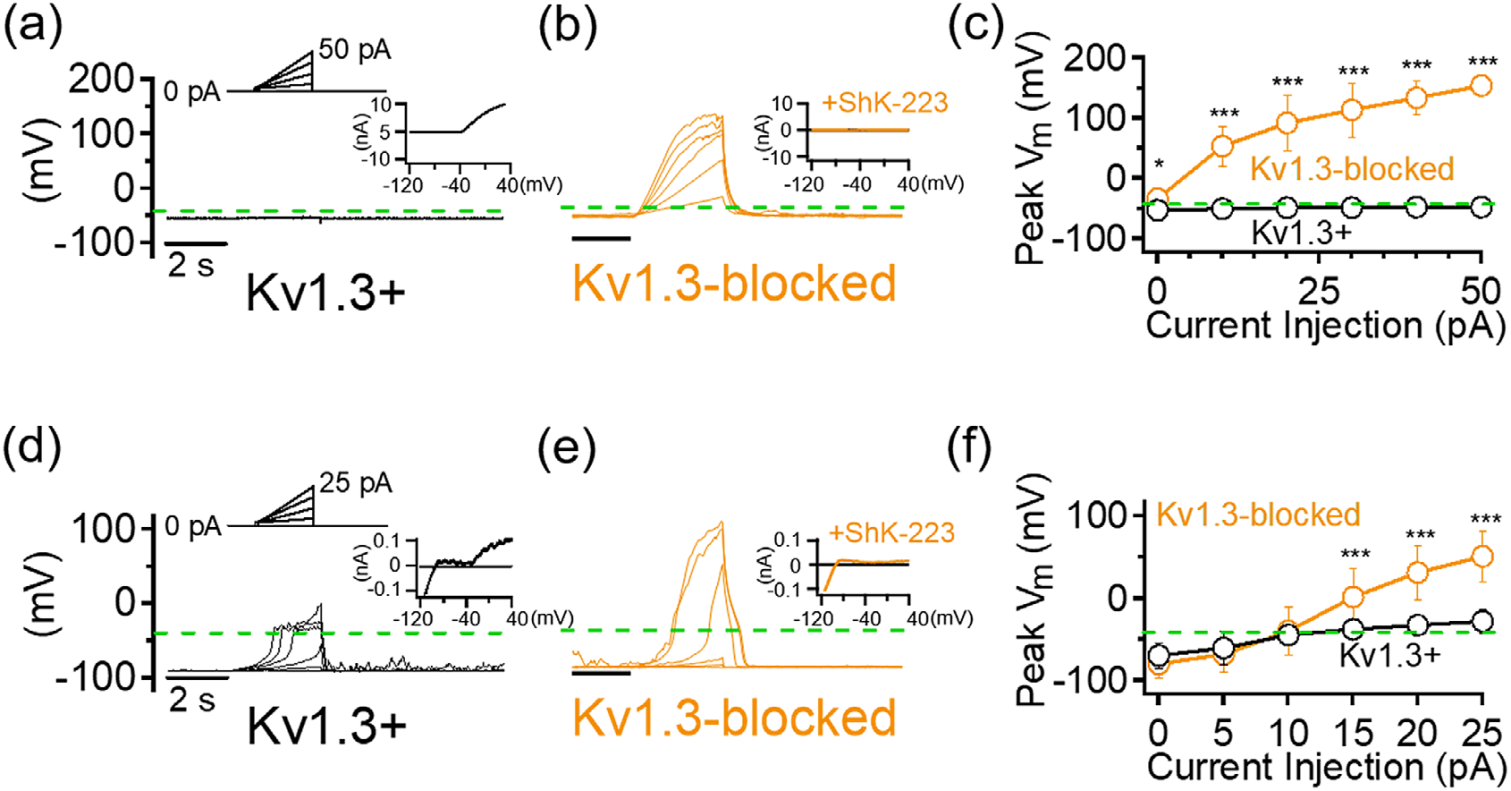
Kv1.3 prevents extreme membrane depolarization triggered by current injections. Sample current‐clamp traces of a Kv1.3+ Chinese Hamster Ovary (CHO) cell before (a) and after (b) 100 nM ShK‐223 (*n* = 8). (Top) Current injection protocol consisted of 3‐s ramps from 0 to 50 pA in 10 pA steps. *Insets*: Voltage‐clamp traces of same cell elicited by a voltage ramp from −120 to +40 mV. (c) Quantification of maximal membrane depolarization measured. Sample current‐clamp traces of Kv1.3+ microglia before (d) and after (e) 100 nM ShK‐223 (*n* = 11). (Top) Current injection protocol consisted of 3‐s ramps from 0 to 25 pA in 5 pA steps. *Insets*: Voltage‐clamp traces of same cell. (f) Quantification of maximal membrane depolarization measured. Dashed green lines indicate the −40‐mV membrane potential level near the Kv1.3 activation threshold potential. Error bars indicate mean ± *SD*. Statistical significance determined by paired *t* test. *p < 0.05, **p < 0.005, and ***p < 0.0005 [Color figure can be viewed at wileyonlinelibrary.com]

Prior to examining the role of Kv1.3 in microglia physiology, we first determined its role in controlling CHO cells membrane potential. The RMP in CHO cells, which lack endogenous voltage‐gated K^+^ channels (Yu & Kerchner, 1998), was relatively depolarized at −23.57 ± 5.58 mV (*n* = 10) before the overexpression of Kv1.3. Cells expressing Kv1.3 at a density of 910.98 ± 550.36 pA/pF (*n* = 6), on the other hand, were found to have membrane potentials of −52.51 ± 4.91 mV (*n* = 6). This hyperpolarizing shift in Kv1.3+ CHO cells has been previously reported (Defarias, Stevens, & Leonard, 1995) and is associated with the efflux of K^+^ ions through Kv1.3 channels activating at potentials near −45 mV and higher when recombinantly expressed (Grissmer et al., 1994). Cells expressing Kv1.3 were also completely resistant to membrane depolarizations evoked by increasing current injections up to 50 pA (Figure 1a,c). In fact, the mean potential evoked by the maximal 50 pA injection is −47.65 ± 2.46 mV (*n* = 6), matching closely the activation threshold potential of Kv1.3 channels. The same current injection paradigm induced robust depolarization in the same cells in the presence of ShK‐223, a selective peptide inhibitor of Kv1.3 (mean peak potential of +154.18 ± 16.81 mV at 50 pA injection, *n* = 8) (Figure 1b,c) and in null CHO cells (data not shown).

We next determined whether the ability of Kv1.3 to clamp evoked potential changes in CHO cells is pertinent to endogenous microglial Kv1.3 channels. Due to their inherently more hyperpolarized RMP compared to CHO cells, current injections resulted in an initial depolarization in undifferentiated microglia that was not observed in null CHO cells (Figure 1d,f). However, this depolarization did not exceed the established activation threshold potential for microglial Kv1.3, which was reported to be slightly positive to −40 mV (Schilling et al., 2000), for the duration of the 3‐s ramp step duration. As observed in Kv1.3‐expressing CHO cells, current injection failed to significantly depolarize Kv1.3+ microglia (Figure 1a,f, mean peak potential of −28.84 ± 13.81 mV at 25 pA injection, *n* = 11). In contrast, microglia blocked with ShK‐223 exhibited excessive depolarization in response to current injection (Figure 1e,f, mean peak potential of +51.21 ± 31.09 mV at 25 pA injection, *n* = 14). Based on these observations, we conclude that one of the roles of Kv1.3 in microglial physiology is “clamping” membrane potential at the channel's activation threshold potential in the event of membrane depolarization.

### 3.2 Relative expression of Kv1.3 and P2X4 in differentially activated microglia in vitro and in vivo

**FIGURE 2:**
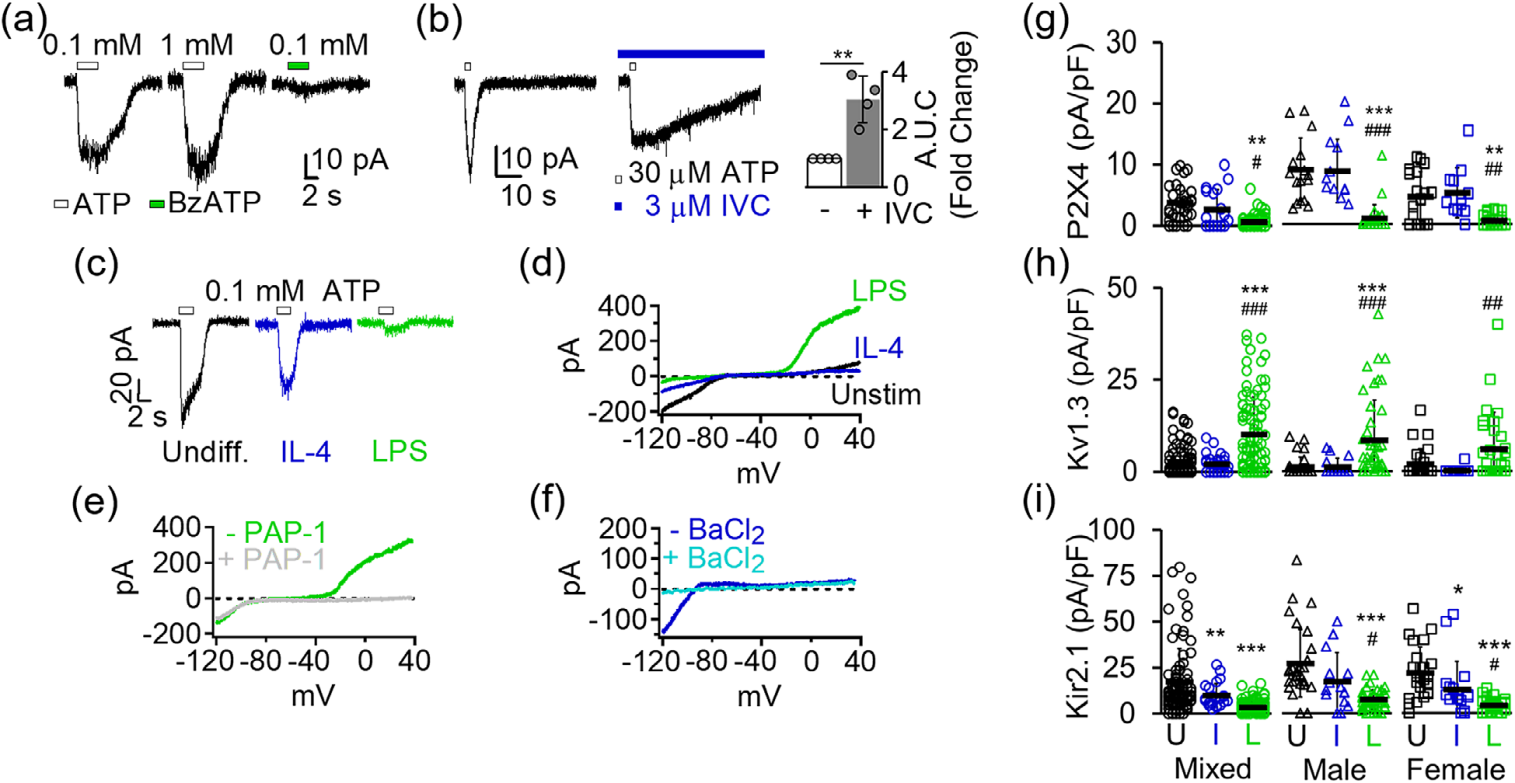
Channel expression changes in differentially activated microglia. (a) Purinergic currents from three representative undifferentiated microglia evoked by a 3‐s pulse of either ATP or BzATP while voltage clamped at −70 mV. (b) Potentiation of ATP‐induced currents by the P2X4‐selective positive modulator ivermectin (*left*). Quantification of area under the curve (AUC) showed a 3.04 ± 0.80‐fold increase in potentiation (*n* = 4) currents induced by 0.03 mM ATP by in the presence of ivermectin (*right*). Statistical significance (**p < 0.01) determined by paired *t* test. (c) Differential P2X4 current expression in undifferentiated, interleukin‐4 (IL‐4), and lipopolysaccharides (LPS)‐differentiated microglia. (d) Overlay of representative K^+^ currents in undifferentiated, IL‐4‐ and LPS‐differentiated microglia. (e) Inhibition of delayed rectifying outward K^+^ current in LPS‐differentiated microglia by 100 nM PAP‐1, a Kv1.3‐selective small molecule blocker. (f) Inhibition of Kir2.1 inward rectifying current by 100 μM BaCl_2_. Scatterplots of (g) P2X4, (h) Kv1.3, and (i) Kir2.1 current density from undifferentiated (U), IL‐4 (I), and LPS‐differentiated (L) cells. Data collected from at least three independently prepared, mixed‐gender, male‐only, and female‐only microglia cultures. Error bars indicate mean ± *SD*. Statistical significance determined by one‐way analysis of variance (ANOVA) followed by Tukey–Cramer's *post hoc* test (alpha = .*p* < 0.05, **p < 0.01, and ***p < 0.001 versus undifferentiated microglia. ^#^ *p* < 0.05, ^##^ *p* < 0.01, ^###^ *p* < 0.001 versus IL‐4 differentiated microglia. See Table 1 for details [Color figure can be viewed at wileyonlinelibrary.com]

Changes to the brain environment during hypoxia and ischemia activate microglia through factors such as ATP released from damaged neurons. We assessed the expression of Kv1.3 relative to that of the P2X4 and P2X7 receptors, two major microglial non‐selective cationic P2X receptors gated by extracellular ATP that conduct mainly Ca^2+^ and can significantly depolarize the microglial membrane (Visentin, Renzi, Frank, Greco, & Levi, 1999). Based on their differential sensitivity to nucleotide derivatives (Chessell et al., 1998; Townsend‐Nicholson, King, Wildman, & Burnstock, 1999), P2X4 and P2X7 currents were detected as inward currents elicited by a saturating concentration of their selective agonist, ATP for P2X4 and BzATP for P2X7, respectively. From mixed‐gender cultures, 0.1 mM ATP induced robust desensitizing inward currents (3.71 ± 2.90 pA/pF, *n* = 37) in undifferentiated microglia, while 0.1 mM BzATP produced very little to no currents (0.09 ± 0.31 pA/pF, *n* = 19) (Figure 2a, Table 1). Then, 1 mM ATP, the minimum concentration required to activate the low‐affinity P2X7 receptor, failed to further increase the amplitude of currents evoked by 0.1 mM ATP (Figure 2a). P2X4 is selectively potentiated by ivermectin (Khakh, Proctor, Dunwiddie, Labarca, & Lester, 1999; Toulme, Soto, Garret, & Boue‐Grabot, 2006). To increase the dynamic range in measuring the effect of ivermectin on P2X4 currents, we used a subsaturating concentration of 0.03 mM ATP and observed a 3.04 ± 0.80‐fold (*n* = 4) increase in the presence of 3 μM ivermectin (Figure 2b), confirming P2X4 as the predominant P2X current in our postnatal cultured microglia.

**TABLE 1:** Electrophysiological voltage‐clamp measurements determining functional Kv1.3, Kir2.1, P2X4, and P2X7 expression in cultured neonatal mouse microglia

Cultures	Stimulus		Kv1.3		Kir2.1		P2X4		P2X7	
		Capacitance (pF)	Current (pA)	Density (pA/pF)	Current (pA)	Density (pA/pF)	Current (pA)	Density (pA/pF)	Current (pA)	Density (pA/pF)
Mixed gender	Undifferentiated	13.44 ± 5.16 (*n* = 97)	32.15 ± 50.50 (*n* = 97)	2.68 ± 4.11 (*n* = 97)	267.98 ± 368.66 (*n* = 97)	17.26 ± 18.17 (*n* = 97)	51.67 ± 39.42 (*n* = 37)	3.71 ± 2.90 (*n* = 37)	1.05 ± 3.15 (*n* = 19)	0.09 ± 0.31 (*n* = 19)
	IL‐4	15.88 ± 4.23 (*n* = 35)	30.23 ± 32.22 (*n* = 30)	2.09 ± 2.44 (*n* = 30)	282.59 ± 233.39 (*n* = 35)	9.99 ± 6.46 (*n* = 35) *p < 5 × 10^−3^	38.66 ± 45.82 (*n* = 18)	2.65 ± 3.27 (*n* = 18) *p < 5 × 10^−2^	3.33 ± 6.51 (*n* = 12)	0.25 ± 0.49 (*n* = 12)
	LPS	18.86 ± 5.99 (*n* = 116) *p < 5 × 10^−11^ ^#^ *p* < 1 × 10^−2^	227.13 ± 253.73 (*n* = 114) *p < 1 × 10^−12^ ^#^ *p* < 1 × 10^−12^	10.16 ± 10.16 (*n* = 114) *p < 1 × 10^−10^ ^#^ *p* < 1 × 10^−11^	65.28 ± 59.31 (*n* = 114) *p < 1 × 10^−6^ ^#^ *p* < 5 × 10^−6^	3.55 ± 3.27 (*n* = 114) *p < 1 × 10^−10^	11.22 ± 17.72 (*n* = 72) *p < 5 × 10^−3^ ^#^ *p* < 5 × 10^−2^	0.63 ± 0.99 (*n* = 72) *p < 5 × 10^−3^ ^#^ *p* < 5 × 10^−2^	3.44 ± 6.74 (*n* = 38)	0.14 ± 0.29 (*n* = 38)
Female	Undifferentiated	8.93 ± 2.88 (*n* = 28)	17.50 ± 42.21 (*n* = 28)	1.95 ± 4.08 (*n* = 28)	196.42 ± 151.51 (*n* = 28)	21.85 ± 14.06 (*n* = 28)	35.78 ± 34.69 (*n* = 21)	4.50 ± 4.04 (*n* = 21)	n.d.	n.d.
	IL‐4	10.64 ± 3.36 (*n* = 18)	2.77 ± 11.78 (*n* = 18)	0.18 ± 0.78 (*n* = 18)	150.55 ± 196.00 (*n* = 18)	12.78 ± 15.43 (*n* = 18) *p < 5 × 10^−2^	50.41 ± 51.41 (*n* = 12)	5.13 ± 4.08 (*n* = 12)	n.d.	n.d.
	LPS	17.20 ± 5.28 (*n* = 39) *p < 5 × 10^−11^ ^#^ *p* < 1 × 10^−6^	91.32 ± 159.46 (*n* = 38) *p < 1 × 10^−2^^#^ *p* < 5 × 10^−3^	6.00 ± 10.04 (*n* = 38) ^#^ *p* < 5 × 10^−3^	63.84 ± 66.47 (*n* = 39) *p < 5 × 10^−4^	4.15 ± 4.15 (*n* = 39) *p < 5 × 10^−7^ ^#^ *p* < 5 × 10^−2^	7.50 ± 13.04 (*n* = 30) *p < 5 × 10^−3^ ^#^ *p* < 5 × 10^−2^	0.54 ± 0.86 (*n* = 30) *p < 5 × 10^−3^ ^#^ *p* < 5 × 10^−3^	n.d.	n.d.
Male	Undifferentiated	8.76 ± 3.05 (*n* = 33)	8.75 ± 19.46 (*n* = 32)	1.18 ± 2.66 (*n* = 32)	232.15 ± 147.52 (*n* = 33)	27.09 ± 18.37 (*n* = 33)	75.00 ± 66.80 (*n* = 17)	8.28 ± 5.22 (*n* = 17)	n.d.	n.d.
	IL‐4	7.51 ± 2.62 (*n* = 18)	12.22 ± 22.21 (*n* = 18)	1.23 ± 2.19 (*n* = 18)	127.22 ± 113.64 (*n* = 18) *p < 5 × 10^−2^	17.08 ± 15.81 (*n* = 18)	58.57 ± 26.56 (*n* = 14)	8.63 ± 5.14 (*n* = 14)	n.d.	n.d.
	LPS	14.69 ± 4.99 (*n* = 46) *p < 5 × 10^−8^ ^#^ *p* < 5 × 10^−9^	118.64 ± 165.80 (*n* = 44) *p < 1 × 10^−4^ ^#^ *p* < 5 × 10^−4^	8.45 ± 10.91 (*n* = 44) *p < 1 × 10^−4^ ^#^ *p* < 5 × 10^−4^	100.88 ± 68.68 (*n* = 44) *p < 5 × 10^−5^	7.38 ± 5.22 (*n* = 44) *p < 1 × 10^−6^ ^#^ *p* < 5 × 10^−2^	12.93 ± 36.29 (*n* = 29) *p < 5 × 10^−3^ ^#^ *p* < 5 × 10^−4^	0.90 ± 2.23 (*n* = 29) *p < 5 × 10^−5^ ^#^ *p* < 1 × 10^−4^	n.d.	n.d.

*Note: n*, number of individual cells combined from three or more separate microglia cultures (N). All data represented as mean ± *SD*; statistical significance among different stimulation conditions (undifferentiated, IL‐4‐treated, and LPS‐treated cells) within or between groups (mixed‐gender, female, or male microglia) determined by one‐way ANOVA with post hoc Tukey–Kramer test (alpha = 0.05). Statistical significance defined as *p* < 5 × 10^−2^ for 1). * signifies direct comparison against undifferentiated cells or 2); ^#^ signifies direct comparison against IL‐4 differentiated microglia; n.d., data not determined.

Abbreviations: ANOVA, analysis of variance; IL‐4, interleukin‐4; LPS, lipopolysaccharides.

Differentiation with IL‐4 had no significant effect on P2X4 expression (Figure 2c,g, Table 1; 2.65 ± 3.27 pA/pF, *n* = 18) nor P2X7 expression (Table 1; 0.25 ± 0.49 pA/pF, *n* = 12). LPS stimulation for 24 hr had no effect on P2X7 current density (0.14 ± 0.29 pA/pF, *n* = 38) but significantly reduced P2X4 current density (0.63 ± 0.99 pA/pF, *n* = 72) by more than 13‐fold (Figure 2c,g). Since P2X4 has been suggested to play a role in the sexual dimorphism of many microglia‐mediated inflammatory functions (Mapplebeck, Beggs, & Salter, 2016) we also quantified its expression changes in microglia that were prepared and cultured separately from male and female postnatal mice. However, P2X4 expression changes largely mirrored changes observed for mixed‐gender microglia and exhibit no significant sex differences between males in female microglia in vitro (Figure 2g and Table 1).

To validate our differentiation method and detect the extent of Kv1.3's coexpression with P2X4 in our system, we also quantified Kv1.3 and Kir2.1 currents, defined as PAP‐1‐sensitive voltage‐gated outwardly rectifying (Figure 2e) and BaCl_2_‐sensitive inwardly rectifying currents (Figure 2f), respectively. From our mixed‐gender microglia cultures, undifferentiated cells displayed low Kv1.3 (2.68 ± 4.11 pA/pF, *n* = 97) and high Kir2.1 (17.26 ± 18.17 pA/pF, *n* = 97) current expression (Figure 2d,f,h,i, Table 1). IL‐4 treatment did not increase Kv1.3 expression (Figure 2d,h, Table 1; 2.09 ± 2.44 pA/pF, *n* = 30) but reduced that of Kir2.1 (Figure 2d,i, Table 1; 9.99 ± 6.46 pA/pF, *n* = 35). LPS stimulation, as expected, drastically increased Kv1.3 by more than threefold (Figure 2d,h, Table 1; 10.16 ± 10.16 pA/pF, *n* = 114) and suppressed Kir2.1 by more than fivefold (Figure 2i, Table 1; 3.35 ± 3.27 pA/pF, *n* = 114). Taken together, these results from 24‐hr in vitro differentiation closely recapitulated our previously observed Kv1.3 and Kir2.1 expression profile from 40 to 48 hr cultures (Nguyen et al., 2017) and did not indicate any gender‐dependent expression pattern for Kv1.3 or Kir2.1 in microglia cultures.

**FIGURE 3:**
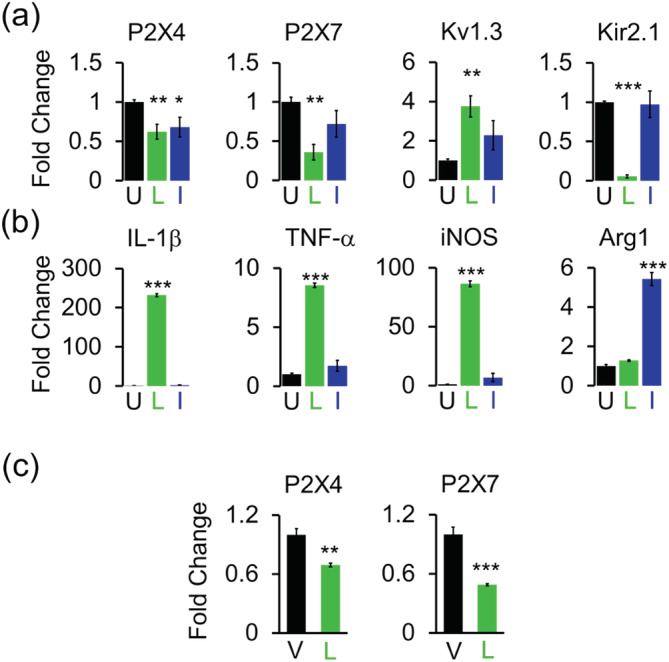
Gene expression changes in differentially activated microglia. Quantitative PCR (qPCR) quantification of mRNA expression of (a) channels and (b) microglia‐associated cytokines and markers in undifferentiated (U), lipopolysaccharides (LPS) (L), and interleukin‐4 (IL‐4) (I) differentiated microglia. Data from three independent mixed‐gender microglia cultures. (c) Quantification of mRNA in acutely isolated CD11b^+^ microglia from mice receiving intracerebroventricular (ICV)‐PBS vehicle (V; *n* = 4) and ICV‐LPS (L; *n* = 4). Bar graphs represent means ± *SEM*. Statistical analysis was performed using unpaired *t* test. *p < 0.05, **p < 0.01, and ***p < 0.001 versus undifferentiated or vehicle‐injected microglia [Color figure can be viewed at wileyonlinelibrary.com]

We confirmed the expression of P2X4 and P2X7 by qPCR to ensure that the observed reduction in channel activity is indeed mirrored by a reduction in mRNA and is not due to differential trafficking to the membrane since these receptors are known to be cycled between the plasma membrane and intracellular compartments (Suurvali, Boudinot, Kanellopoulos, & Ruutel Boudinot, 2017; Xu et al., 2014). LPS stimulation of mixed‐gender microglia cultures for 24 hr suppressed the transcripts for P2X4, P2X7, and Kir2.1 (Figure 3a), consistent with the reduction in current expression measured by electrophysiology. In contrast, LPS increased mRNA expression for Kv1.3 together with that of the M1 pro‐inflammatory markers IL‐1β, TNF‐α, and iNOS (Figure 3a) as we previously described (Di Lucente et al., 2018; Nguyen et al., 2017). Transcripts for these channels did not significantly change in IL‐4 differentiated cells except for that of P2X4 (Figure 3a). IL‐4 increased mRNA for the M2 marker arginase 1 (Figure 3b).

We previously confirmed that the in vitro observed LPS induced Kv1.3 upregulation (Nguyen et al., 2017) also takes place in vivo by investigating Kv1.3 expression in microglia acutely isolated with anti‐CD11b^+^‐magnetic beads 24 hr after ICV‐LPS using qPCR and electrophysiology (Di Lucente et al., 2018). In the current study, we also found that the in vitro observed LPS‐induced reduction in P2X4 and P2X7 gene expression (Figure 3a) occurs in vivo in microglia isolated from ICV‐LPS brains (Figure 3c).

### 3.3 Kv1.3 channel activity influences P2X4‐mediated membrane depolarization

**FIGURE 4 1:**
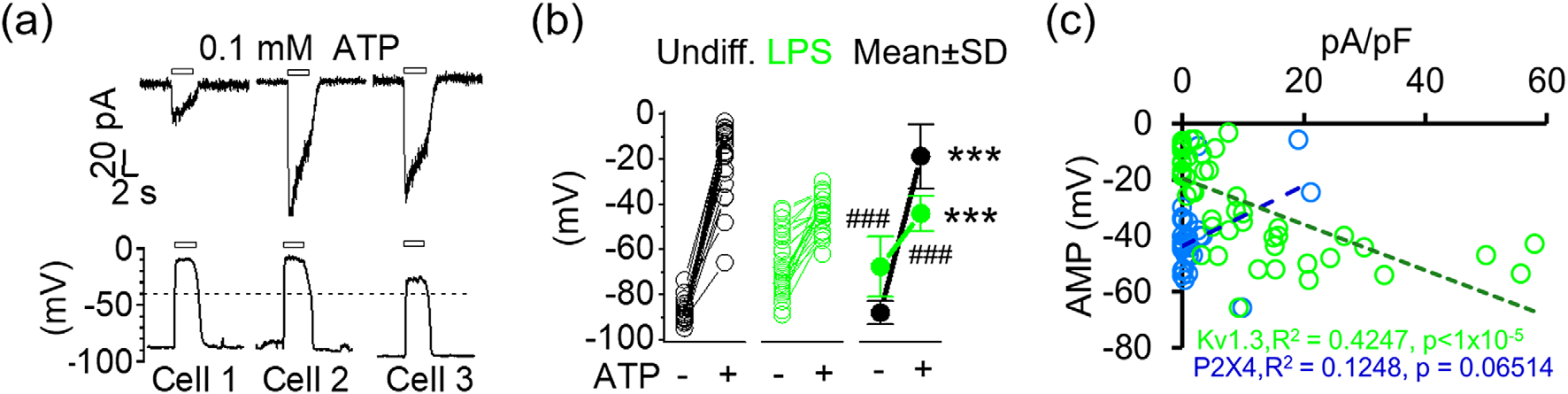
Influence of Kv1.3 expression on membrane potential changes. (a) Corresponding voltage‐clamp (*top*) and current‐clamp (*bottom*) traces induced by 0.1 mM ATP from three individual microglia. (b) Scatterplots for resting membrane potential (RMP) and ATP‐induced membrane potential (AMP). RMP's measured in undifferentiated cells and lipopolysaccharides (LPS)‐differentiated cells averaged −88.08 ± 5.14 mV (*n* = 27) and −67.64 ± 12.62 mV (*n* = 28), respectively. AMP's in undifferentiated cells and LPS‐differentiated cells averaged −19.32 ± 14.49 mV and −44.09 ± 7.67 mV, respectively. Data represented by means ± *SD*. Statistical significance between before and after ATP addition determined by paired *t* test and between undifferentiated and lipopolysaccharides (LPS)‐differentiated microglia determined by one‐way analysis of variance (ANOVA) followed by post hoc Tukey–Cramer's test. ***p < 0.001 denotes significance versus before ATP, ^###^ *p* < 0.001 denotes significance versus undifferentiated cells. See Table 2 for detailed measurements. (c) Pearson correlation between AMP and current density for P2X4 (blue) and Kv1.3 (green). Correlation coefficient, *r*, calculated from a total of 52 control undifferentiated and 20 LPS‐treated cells. Statistical significance is set at *p* < 0.05 [Color figure can be viewed at wileyonlinelibrary.com]

Activation of P2X4 receptors by 0.1 mM ATP has been reported to result in massive membrane depolarizations that can reach close to 0 mV (Visentin et al., 1999). Based on our observation that Kv1.3 channel activity prevents excessive depolarization invoked by current injection, we hypothesized that Kv1.3 channels could render P2X4‐expressing microglia resistant to membrane depolarization triggered by ATP. Current‐clamp recordings performed on P2X4‐expressing microglia showed that membrane depolarizations elicited by 0.1 mM ATP were mostly robust and independent of the amplitude of ATP‐gated P2X4 currents (Figure 4a). Undifferentiated cells overall exhibit a hyperpolarized RMP (−88.19 ± 5.07 mV, *n* = 28) in comparison with LPS‐differentiated cells (−67.64 ± 13.36 mV, *n* = 28, *p* < 1 × 10^−8^). However, AID in undifferentiated microglia (68.87 ± 15.65 mV, *n* = 28) was strikingly larger than in LPS‐differentiated cells (23.54 ± 11.16 mV, *n* = 28, *p* < 1 × 10^−16^) (Figure 4b and Table 2). As a result, undifferentiated cells, despite exhibiting a hyperpolarized RMP, showed an ATP‐induced membrane potential (AMP) that is much more depolarized (−19.32 ± 14.49 mV, *n* = 28) than the membrane potential of LPS‐differentiated cells (−44.10 ± 7.68 mV, *n* = 28, *p* < 1 × 10^−9^) (Figure 4b and Table 2). Although, these results suggest a significant dependency of AID and, hence AMP, on the presence of Kv1.3, there exists a possible contributing role for P2X4 since its downregulation by LPS means reduced net calcium influx. However, when we plotted the individual AMPs against the corresponding current densities of P2X4 and Kv1.3 to determine their relative contribution to AMP, we found that microglial AMP correlates better with Kv1.3 expression than with P2X4 expression. The calculated Pearson correlation coefficient, *r*, between Kv1.3 expression and AMP is −0.666 (*R* ^2^ = 0.4347, *p* < 5 × 10^−5^), while that of P2X4 expression and AMP is 0.36 (*R* ^2^ = 0.1248, *p* = 0.6) (Figure 4c), indicating that Kv1.3 plays a more substantial role than P2X4 in determining microglia membrane potential changes.

**TABLE 2:** Kv1.3 regulates microglial RMP and P2X4‐mediated membrane potential changes

Treatments	RMP (mV)	AMP (mV)	AID (mV)
Undifferentiated	−88.19 ± 5.08 (*n* = 28)	−19.32 ± 14.49 (*n* = 28) *p < 1 × 10^−18^	68.87 ± 15.65 (*n* = 28)
LPS	−67.64 ± 13.36 (*n* = 28) ^#^ *p* < 1 × 10^−8^	−44.10 ± 7.68 (*n* = 28) *p < 5 × 10^−4^ ^#^ *p* < 1 × 10^−9^	23.54 ± 11.16 (*n* = 28) ^#^ *p* < 1 × 10^−16^

*Note:* Effects of Kv1.3 expression on RMP, AMP by 100 μM ATP, and AID. “*n*,” number of individual cells from three or more separate microglia cultures. All data represented as mean ± *SD*. Statistical significance for comparison between before and after ATP (*) was determined by paired *t* test. Statistical significance for comparison between undifferentiated and LPS‐differentiated microglia (^#^) was determined by one‐way ANOVA followed by post hoc Tukey–Cramer's test. Statistical significance defined as *p* < 5 × 10^−2^.

Abbreviations: AID, ATP‐induced depolarization; AMP, ATP‐induced membrane potential; ANOVA, analysis of variance; LPS, lipopolysaccharides; RMP, resting membrane potential.

**FIGURE 5:**
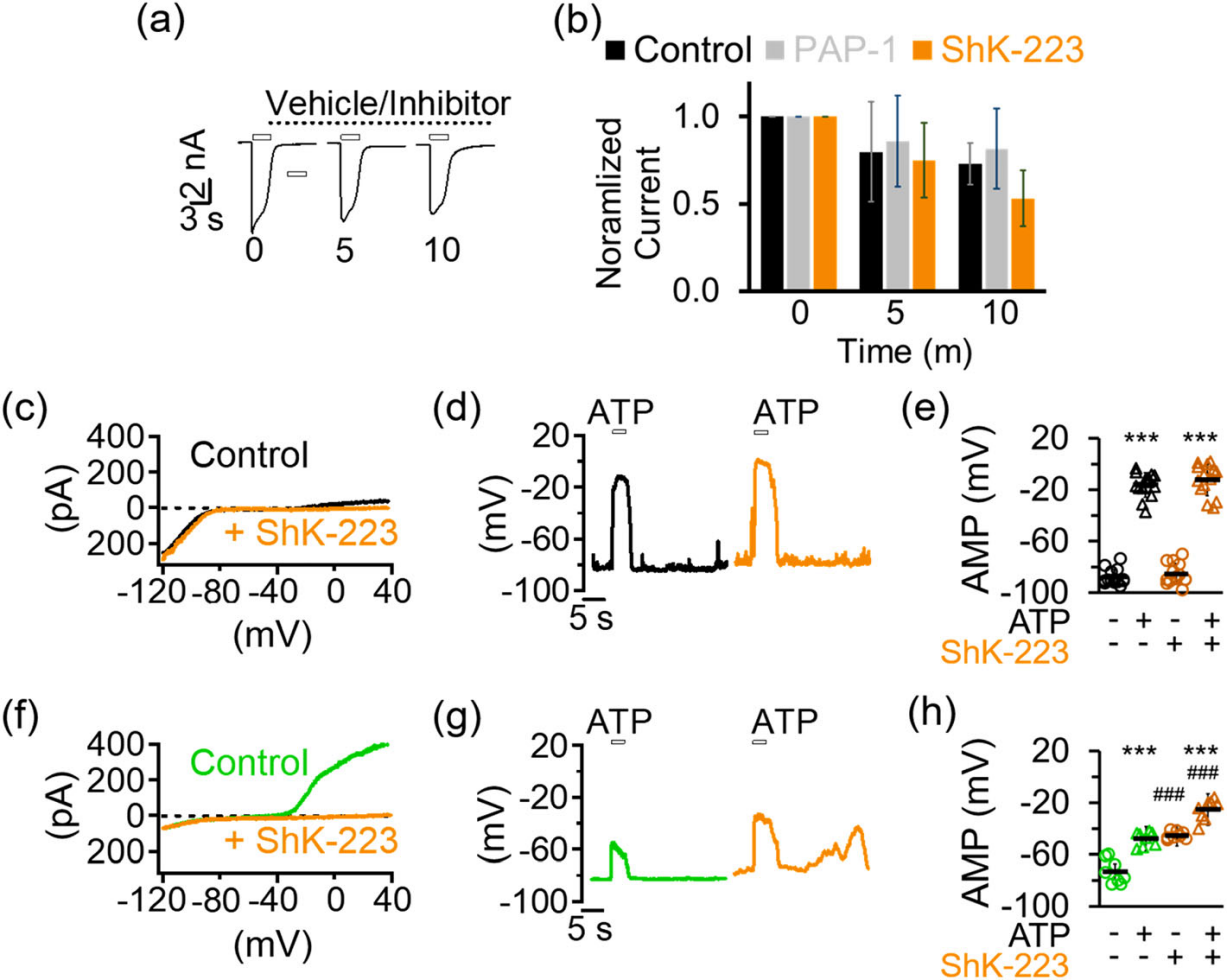
Kv1.3 blockade depolarizes microglia and disrupts resistance to ATP‐induced membrane depolarization. (a) Kv1.3 inhibitors do not cross‐react with P2X4. Sample recording of P2X4 currents elicited by 0.1 mM ATP in a Chinese Hamster Ovary (CHO) cell at the 0, 5, and 10‐min time points displaying characteristic time‐dependent current rundown. (b) Bar graphs showing normalized current for control cells (*n* = 5), PAP‐1 (1 μM) treated cells (*n* = 4), and ShK‐223 (100 nM) treated cells (*n* = 5). Inhibitors were added immediately after the first ATP pulse and remained in the recording chamber throughout the duration between and during subsequent ATP pulses. Error bars denote means ± *SD*. (c) Voltage‐clamp currents before and after inhibition of Kv1.3 with 100 nM ShK‐223 in an undifferentiated microglial cell. (g) Current‐clamp displaying ATP‐induced depolarization (AID) of resting membrane potential (RMP) before and after ShK‐223 in the same undifferentiated cell. (e) Scatterplots summarizing RMP and AMP levels before and after ShK‐223 for undifferentiated cells (*n* = 14). (f) Voltage‐clamp currents before and after inhibition of Kv1.3 with 100 nM ShK‐223 in an lipopolysaccharides (LPS)‐stimulated microglial cell. (g) Current‐clamp displaying AID of RMP before and after ShK‐223 in the same LPS‐stimulated cell. (h) Scatterplots summarizing RMP and AMP levels for LPS‐treated cells (*n* = 8). Statistical significance determined by paired *t* test. ***p < 0.001 annotates significance versus before ATP. ^###^ *p* < 0.001 annotates significance versus before inhibition by ShK‐223. See Table 3 for details [Color figure can be viewed at wileyonlinelibrary.com]

We next used pharmacology to directly confirm the role of Kv1.3 in enabling microglia to resist extreme membrane potential depolarizations. We first showed that ShK‐223, which has been extensively tested for selectivity for Kv1.3 over other K^+^ channels (Pennington et al., 2015), does not cross react to P2X4 channels in recombinant expression systems at 100 nM (Figure 5a,b). Similarly, the small molecule Kv1.3 blocker PAP‐1 also did not inhibit P2X4. Inhibition of Kv1.3 channels (Figure 5c,f), as expected, had a significant effect on microglial RMP and AMP in Kv1.3‐high microglia. In undifferentiated Kv1.3‐low microglia, 100 nM ShK‐223 did not depolarize RMP nor AMP by a significant amount (Figure 5c–e, Table 3). However, 100 nM ShK‐223 had a much more drastic effect on RMP and AMP of LPS‐differentiated cells, which express significantly more Kv1.3 than undifferentiated cells. Then, 100 nM ShK‐223 depolarized RMP by more than 28 mV and AMP by more than 20 mV (Figure 5f–h, Table 3), indicating that blocking Kv1.3 compromised the ability of LPS‐differentiated microglia to resist AID.

**TABLE 3:** Effects of Kv1.3 inhibition on microglial RMP and P2X4‐mediated membrane potential changes

	− ShK‐223			+ ShK‐223		
Treatments	RMP (mV)	AMP (mV)	AID (mV)	RMP (mV)	AMP (mV)	AID (mV)
Undifferentiated	−88.05 ± 6.08 (*n* = 14)	−16.30 ± 9.11 (*n* = 14) *p < 5 × 10^−13^	71.74 ± 9.11 (*n* = 14)	−85.47 ± 8.05 (*n* = 14)	−12.36 ± 12.29 (*n* = 14) *p < 5 × 10^−12^	73.11 ± 10.56 (*n* = 14)
LPS	−73.37 ± 9.34 (*n* = 8)	−47.93 ± 5.52 (*n* = 8) *p < 5 × 10^−4^	25.44 ± 9.35 (*n* = 8)	−45.39 ± 2.93 (*n* = 8) ^#^ *p* < 5 × 10^−12^	−25.36 ± 8.16 (*n* = 8) *p < 5 × 10^−4^ ^#^ *p* < 5 × 10^−5^	20.04 ± 7.54 (*n* = 8)

*Note:* Effects of Kv1.3 inhibition by 100 nM ShK‐223 on RMP, AMP by 100 μM ATP, and AID. “*n*,” number of individual cells from three or more separate microglia cultures. All data represented as mean ± *SD*. Paired *t* test was used to determine statistical significance between before and after ATP (*) or before and after ShK‐223 (^#^). Statistical significance defined as *p* < 5 × 10^−2^.

Abbreviations: AID, ATP‐induced depolarization; AMP, ATP‐induced membrane potential; LPS, lipopolysaccharides; RMP, resting membrane potential.

### 3.4 Kv1.3 drives Ca^2+^ entry across P2X4 receptors

**FIGURE 6:**
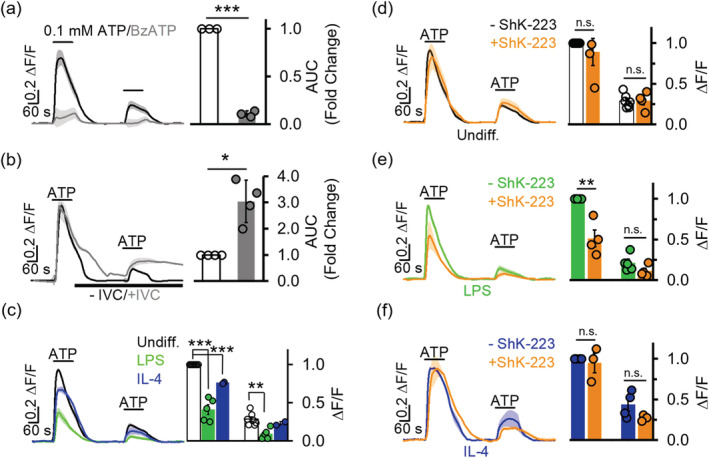
Kv1.3 channel inhibition reduces intracellular Ca^2+^ signaling. (a) Fluo‐4 AM calcium indicator fluorescence signal elicited by 0.1 mM ATP is 2.65 ± 0.99‐fold (*n* = 3) higher in total area under the curve (AUC) compared to that elicited by 0.1 mM BzATP (*n* = 3). Statistical significance determined by unpaired *t* test comparing ATP and BzATP cells. (b) Ivermectin (IVC, 3 μM) increases fluorescence signaling elicited by 0.1 mM ATP by 2.65 ± 0.99‐fold (*n* = 4). Statistical significance between before and after ivermectin determined by paired *t* test. (c) Twenty‐four hours treatment with lipopolysaccharides (LPS) (300 ng/ml) or interleukin‐4 (IL‐4) (20 ng/ml) suppresses fluorescence increase. Statistical significance determined by one‐way analysis of variance (ANOVA) followed by Tukey–Cramer's post hoc test (alpha = 0.05). (d–f) Preincubation with the Kv1.3 blocker ShK‐223 (100 nM) reduces ATP‐mediated fluorescence increases in LPS‐treated microglia but not in undifferentiated or IL‐4 stimulated microglia. All ATP applied at 0.1 mM and after baseline fluorescence was recorded for 2 min. Changes in [Ca^2+^]_i_ are represented as ΔF/F (change in fluorescence measured as AUC after baseline subtraction). Scale bars indicate 20% of the maximal normalized change in ΔF/F, which is 1ΔF/F. Statistical significance determined by paired *t* test. All data presented as mean ± *SEM*. Measurements from three to seven separate experiments (coverslips from different cultures on different days) and 50–100 cells each were measured per experiment for panels (c)–(f). *p < 0.05, **p < 0.01, ***p <* 0.001* [Color figure can be viewed at wileyonlinelibrary.com]

Since Kv1.3 channels exerted a significant influence on microglial membrane potential, we next investigated their role in modulating the driving force behind Ca^2+^ entry triggered by ATP using the fluorescence dye indicator Fluo‐4 AM. Undifferentiated microglia perfused with normal extracellular medium showed a faint but detectable fluorescence during the first 2 min of baseline recording, indicating a low resting [Ca^2+^]_i_. Fluorescence rose quickly to a plateau and then declined in response to a 2‐min application of 0.1 mM ATP, but not 0.1 mM BzATP (Figure 6a). The BzATP‐induced Ca^2+^ signal was less than 10% of that induced by ATP (*p* < 1 × 10^−6^, Figure 6a). A second application of 0.1 mM ATP also induced a significant increase in [Ca^2+^]_i_ but only at a fraction of the initial peak, which is likely due to the characteristic “run‐down” effect resulting from receptor desensitization to ligands for P2X4Rs (North, 2002; Visentin et al., 1999; Walz, Ilschner, Ohlemeyer, Banati, & Kettenmann, 1993). Furthermore, the ATP‐evoked fluorescence increase was positively facilitated by addition of 3 μM of the P2X activator ivermectin to the extracellular medium, which increased total signal by threefold (Figure 6b). Taken together, these results show that Ca^2+^ entry through the P2X4R pore is the dominant ATP‐induced calcium source in cultured neonatal microglia.

Stimulation of microglia with LPS significantly reduced the ATP‐induced Ca^2+^ entry (Figure 6c), confirming the reduction in P2X4 current and mRNA expression we had observed in Figures 2 and 3. Interestingly, stimulation of microglia with IL‐4 lowered Ca^2+^ entry upon stimulation with ATP compared to undifferentiated cells (Figure 6c) but less than LPS stimulation did. Since IL‐4 did not reduce P2X4 current density (Figure 2g), and inhibiting Kv1.3 currents had a major impact on membrane potential (Figure 5), this reduction in Ca^2+^ flux is likely due to the lack of a restoration of the driving force for calcium entry due to the very low levels of Kv1.3 channels in IL‐4 differentiated cells. Accordingly, we predicted that inhibition of Kv1.3 in LPS‐treated cells, which already had a reduced P2X4 expression, should further interfere with the of Ca^2+^ entry through P2X4 receptors. Indeed, fluorescence measurements in LPS‐differentiated cells were attenuated after a 30‐min preincubation with ShK‐223 (Figure 6e) or an acute application of 100 nM PAP‐1 (Figure S1). Similar treatments with ShK‐223 did not alter fluorescence activity in undifferentiated or IL‐4 derived microglia (Figure 6d,f), reflecting the low Kv1.3 expression in these cells.

### 3.5 P2X4 expression changes in ischemic stroke in mice

**FIGURE 7:**
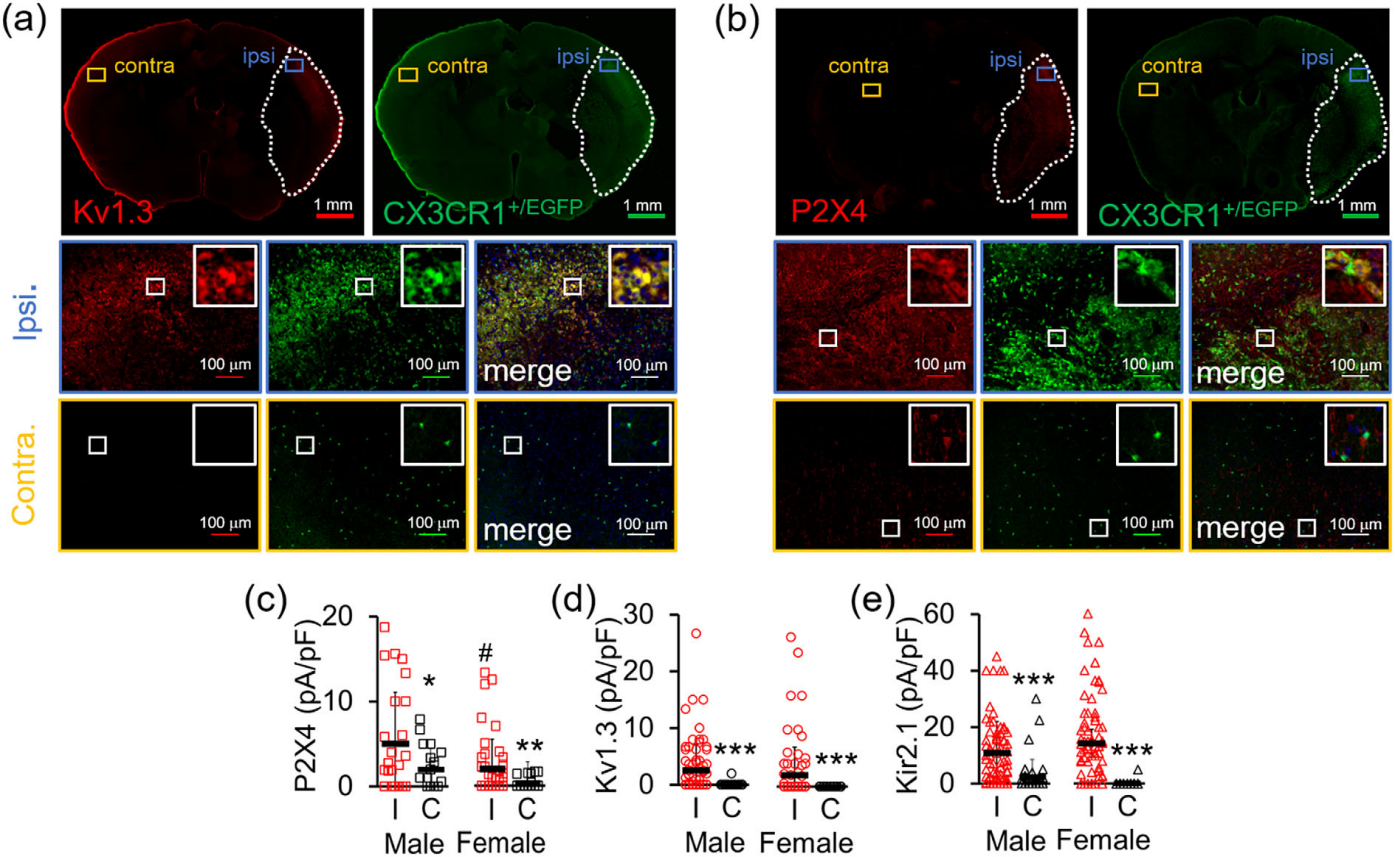
Expression changes of Kv1.3 channels and P2X4 receptors in in microglia isolated from Cx3CR1^+/EGFP^ transgenic mice 8 days after middle cerebral artery occlusion (MCAO) as a model of ischemic stroke. Sample immunofluorescence staining of 14‐μM thick coronal brain sections from the 6‐mm depth showing (a) increased Kv1.3 (*red*) and (b) P2X4 (*red*) immunoreactivity in ipsilateral Cx3CR1^+/EGFP^ (*green*) cells but not contralateral cells. Each channel was analyzed on n = 3–4 coronal sections from N = 3 male and 3 female mice. (c) P2X4 (d) Kv1.3 and (e) Kir2.1 current densities measured from CD11b^+^ Cx3CR1^+/EGFP^ microglia acutely isolated from the ipsilateral hemisphere (8 days after MCAO) compared to microglia isolated from the contralateral side. Statistical significance determined by one‐way analysis of variance (ANOVA) followed by Tukey–Cramer's post hoc (alpha = 0.05). *p < 0.05, **p < 0.01, and ***p < 0.001 versus ipsilateral microglia. ^#^ *p* < 0.05 versus male microglia. See Table 4 for details [Color figure can be viewed at wileyonlinelibrary.com]

Our laboratory had previously reported increased Kv1.3 and Kir2.1 current densities in microglia acutely isolated from the infarcted, so‐called ipsilateral side, compared to microglia isolated from the contralateral side from male mice subjected to MCAO, a model of ischemic stroke (Chen et al., 2016). We here subjected male and female CX_3_CR1^+/EGFP^ transgenic mice to transient MCAO with 8 days of reperfusion in order to investigate P2X4, Kv1.3, and Kir2.1 expression in microglia in both genders (Figure 7). In these mice, where one copy of the chemokine receptor CX_3_CR1 is replaced by EGFP, microglia, and brain infiltrating macrophages are green fluorescent and are therefore easy to identify in tissue sections and after isolation (Jung et al., 2000). Immunofluorescence images of coronal sections prepared from mice sacrificed 8 days after reperfusion showed the expected increase in EGFP fluorescence in the cortex and striatum of the infarcted area (outlined in white dots in Figure 7a,b) due to increased microglia/macrophage activation and infiltration. Kv1.3 and P2X4 immunoreactivity was also increased in the infarcted area and colocalizes largely with CX_3_CR1 in the ipsilateral but not the contralateral side (Figure 7a,b). However, in contrast to Kv1.3 immunoreactivity, which was not observed in noninfarcted tissue, there was some P2X4 expression visible on the contralateral side and in the infarct on cells that are not CX_3_CR1^+^ (Figure 7b). We presume both are due to neuronal P2X4 expression (Stokes, Layhadi, Bibic, Dhuna, & Fountain, 2017).

In order to more “cleanly” investigate microglial ion channel expression and potential gender differences, we acutely isolated CD11b^+^ cells from both male and female CX_3_CR1^+/EGFP^ mice 8 days after MCAO and studied them by whole‐cell patch‐clamp. Contralateral noninfarct microglia from both genders exhibited similar current densities for P2X4, Kv1.3, and Kir2.1 (Figure 7c–e, Table 4). As expected, microglia isolated from the ipsilateral stroke brain are activated in comparison to contralateral control microglia as demonstrated by an approximately two‐fold increase in cell capacitances (Table 4) and displayed significantly higher current amplitudes and densities for P2X4, Kv1.3, and Kir2.1 (Figure 7c–e, Table 4). Interestingly, ipsilateral microglia from male mice exhibited higher P2X4 expression than cells from female mice (Figure 7c, Table 4), while there was no difference in Kir2.1 and Kv1.3 current densities between the sexes following MCAO (Figure 7d,e). This difference in P2X4 response in male versus female has been suggested to play a role in the sexual dimorphism of many microglia‐mediated inflammatory functions (Mapplebeck et al., 2016).

**TABLE 4:** Electrophysiological voltage‐clamp measurements of acutely isolated CD11b^+^ microglia from ischemic MCAO mouse brain

Gender			P2X4		Kir2.1		Kv1.3	
		Capacitance (pF)	Current (pA)	Density (pA/pF)	Current (pA)	Density (pA/pF)	Current (pA)	Density (pA/pF)
Male	Ipsilateral	6.77 ± 3.19 (*n* = 77)	48.00 ± 60.67 (*n* = 26)	5.03 ± 6.02 (*n* = 26)	82.53 ± 97.18 (*n* = 77)	10.92 ± 11.13 (*n* = 77)	14.61 ± 21.71 (*n* = 77)	2.56 ± 4.56 (*n* = 77)
	Contralateral	3.00 ± 1.86 (*n* = 42) *p < 5 × 10^−10^	4.28 ± 4.26 (*n* = 21) *p < 5 × 10^−3^	1.98 ± 246 (*n* = 21) *p < 5 × 10^−2^	5.83 ± 14.8 (*n* = 42) *p < 5 × 10^−9^	2.43 ± 6.09 (*n* = 42) *p < 5 × 10^−7^	0.12 ± 0.77 (*n* = 42) *p < 5 × 10^−7^	0.05 ± 0.30 (*n* = 42) *p < 1 × 10^−5^
Female	Ipsilateral	6.11 ± 3.41 (*n* = 82)	13.95 ± 20.70 (*n* = 44) ^#^ *p* < 2 × 10^−5^	2.02 ± 3.47 (*n* = 44) ^#^ *p* < 5 × 10^−2^	94.82 ± 114.47 (*n* = 82)	14.26 ± 14.19 (*n* = 82)	11.54 ± 34.15 (*n* = 81)	1.98 ± 4.90 (*n* = 81)
	Contralateral	3.13 ± 1.88 (*n* = 34) *p < 1 × 10^−5^	1.04 ± 1.94 (*n* = 24) *p < 5 × 10^−4^	0.33 ± 0.63 (*n* = 24) *p < 2 × 10^−3^	0.29 ± 1.71 (*n* = 34) *p < 1 × 10^−10^	0.14 ± 0.86 (*n* = 34) *p < 5 × 10^−13^	0.00 ± 0.00 (*n* = 34) * *p* < 5 × 10^−3^	0.00 ± 0.00 (*n* = 34) *p < 5 × 10^−4^

*Note:* “*n*,” combined number of individual cells from three or more animals. All data represented as mean ± *SD*; statistical significance determined by one‐way ANOVA followed by Tukey–Cramer's post hoc (alpha = 0.05). “*” versus ipsilateral microglia. “^#^” versus male microglia.

Abbreviations: ANOVA, analysis of variance; MCAO, middle cerebral artery occlusion.

### 3.6 Kv1.3 inhibition prevents microglia activation into a pro‐inflammatory subtype in vitro

**FIGURE 8:**
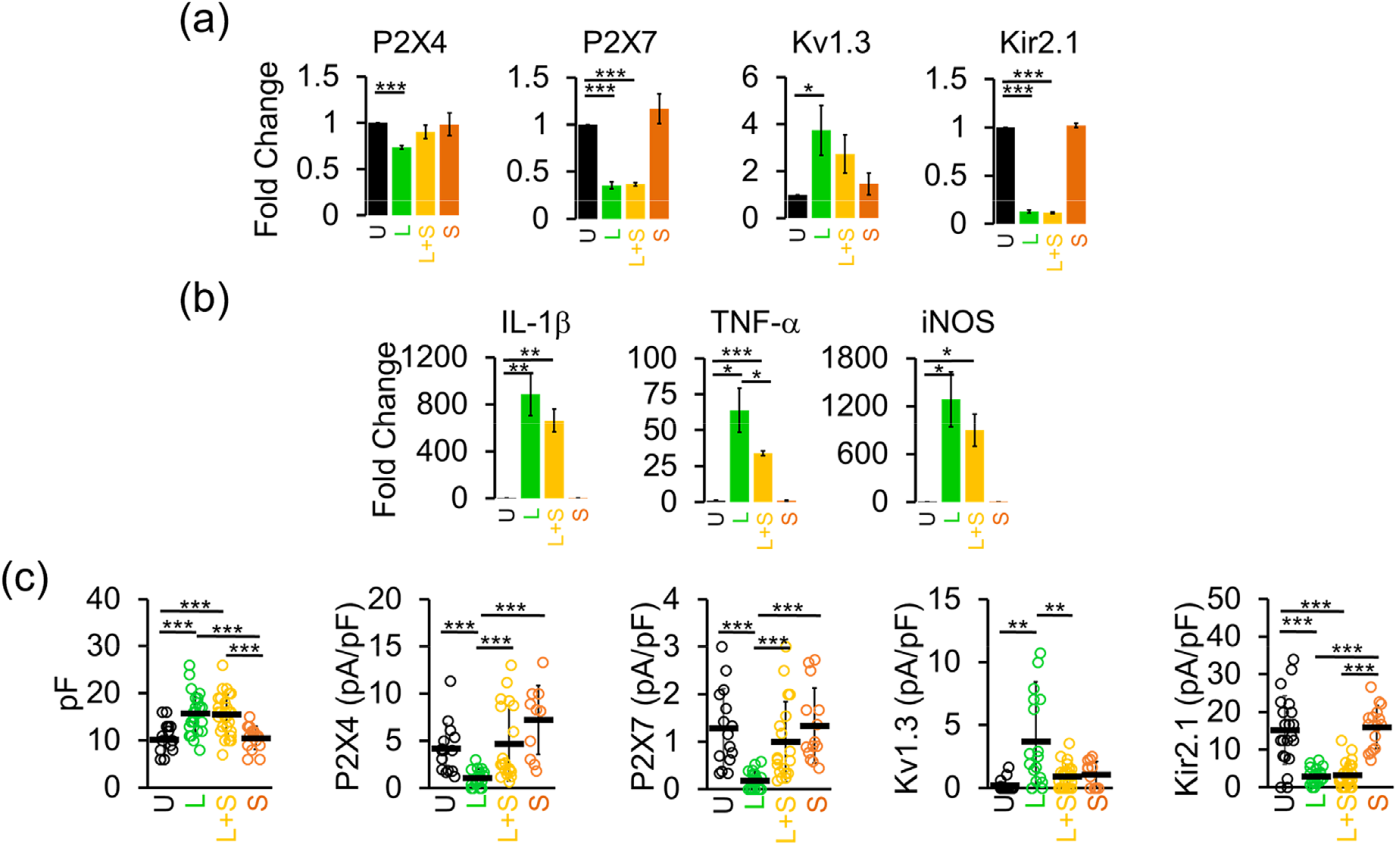
Effects of Kv1.3 channel inhibition on mRNA expression of microglial channels and pro‐inflammatory cytokines. Quantitative PCR (qPCR) quantification of mRNA expression of (a) channels and (b) microglia‐associated cytokines in undifferentiated (U), lipopolysaccharides (LPS) only (L; 300 ng/ml), LPS + 100 nM ShK‐223 (L + S) and 100 nM ShK‐223 only (S) treated microglia. Data from three independent mixed‐gender postnatal microglia cultures. Bar graphs represent means ± *SEM*. Statistical analysis was performed using unpaired *t* test. *p <0.05, **p <0.01, and ***p <0.001 versus undifferentiated microglia. (c) Scatterplots of cell capacitance, P2X4, P2X7, Kv1.3, and Kir2.1 current density undifferentiated (U; *n* = 30), LPS only (L; 300 ng/ml; *n* = 16), LPS + 100 nM ShK‐223 (L + S; *n* = 30), and 100 nM ShK‐223 only (S; *n* = 24) treated microglia. Data collected from at least three independently prepared, mixed‐gender cultures and error bars indicate mean ± *SD*. Statistical significance determined by one‐way analysis of variance (ANOVA) followed by Tukey–Cramer's post hoc test (alpha = 0.05). *p < 0.05, **p <0.01, and ***p < 0.001 [Color figure can be viewed at wileyonlinelibrary.com]

We next asked whether Kv1.3 inhibition would reduce P2X4 expression. In parallel to determining the effect of ShK‐223 on P2X4 expression, we also examined its effect on the mRNA and functional expression of other channels. In brief, while blocking Kv1.3 with ShK‐223 significantly prevented the LPS‐induced downregulation and upregulation of P2X4 and Kv1.3 membrane current density, respectively (Figure 8c), it did not affect the increase in cell size as measured in pF or suppression of Kir2.1 current density (Figure 8c). These changes were paralleled by changes in mRNA expression of these channels (Figure 8a). Interestingly, ShK‐223 also prevented the reduction of P2X7 current expression but had no effect on P2X7 mRNA, suggesting that Kv1.3 is not involved in the transcriptional regulation of this receptor. Overall, our data suggest that Kv1.3 channel function is required for the polarization of activated microglia into a specific subset but not for microglia activation per se since it did not prevent the increase in cell size. Based on the finding that ShK‐233 significantly reduced TNF‐α mRNA and induced a downward trend in IL‐1β and iNOS mRNA expression at 24 hr (Figure 8b), we conclude that Kv1.3 is specifically required for the polarization of activated microglia into a pro‐inflammatory state. These results are in line with our previously published work showing that Kv1.3 inhibition is beneficial in alleviating pro‐inflammatory microglia responses (Chen et al., 2018; Maezawa et al., 2018; Nguyen et al., 2017).

## 4 DISCUSSION

Microglia‐mediated inflammation is an innate immune response in the CNS that can exacerbate neurodegeneration. Expression of the voltage‐gated Kv1.3 channel is increased in activated microglia associated with ischemic stroke, AD, multiple sclerosis and radiation induced damage (Chen et al., 2016; Chen et al., 2018; Maezawa et al., 2018; Peng et al., 2014; Rangaraju et al., 2015; Rus et al., 2005). The ion channel activity of Kv1.3 seems to be a prerequisite for microglia activation as both genetic deletion and pharmacological blockade of Kv1.3 diminished microglial activation and concomitant inflammatory responses, leading to improved pathological and neurological outcomes in several animal models of neuroinflammation (Chen et al., 2018; Di Lucente et al., 2018; Maezawa et al., 2018). Here, we report that the mechanism underlying the effectiveness of Kv1.3 inhibition in alleviating microglia‐mediated neuroinflammation involves, in part, a diminished calcium signaling mediated by the purinergic P2X4 receptor.

Repolarizing outward currents carried by Kv1.3 channels can influence the driving force for Ca^2+^ entry as demonstrated by the observation that overexpression of Kv1.3 in CHO cells lowers the RMP to the channel's activation threshold level. Similarly, blockade of Kv1.3 with ShK‐223 depolarizes Kv1.3‐expressing microglia. These findings corroborate the previously reported role of Kv1.3 in maintaining the negative RMP required for Ca^2+^ influx upon T‐cell receptor activation in human and mini‐swine T‐cells (Cahalan & Chandy, 2009; Koo et al., 1997; Leonard et al., 1992; Verheugen, Vijverberg, Oortgiesen, & Cahalan, 1995). However, in contrast to T‐cells, the additional presence of Kir2.1 channels (Eder, 1998; Kettenmann et al., 2011) in microglia can also influence microglial RMP. Confirming our previous reports (Nguyen et al., 2017) we showed here that stimulation with LPS transforms undifferentiated Kir2.1^high^Kv1.3^low^ cultured neonatal mouse microglia into Kir2.1^low^Kv1.3^high^ microglia. Correspondingly, RMP measured in undifferentiated cells ranges from −73 to −95 mV, with a mean of −88.90 mV that was shifted to −66.26 mV after LPS treatment. This depolarizing shift in response to LPS is likely due to a reduction in Kir2.1 channel expression since inward rectifying Kir channels can only conduct inward K^+^ currents at potentials negative to the Nernst potential for K^+^ ions to restore membrane potential back to the reversal potential for K^+^ ions (Hibino et al., 2010; Sakmann & Trube, 1984). Indeed, it has been shown that blocking Kir2.1 with Ba^2+^ depolarizes LPS‐differentiated rat microglia (Chung, Jung, & Lee, 1999; Franchini, Levi, & Visentin, 2004). Kv1.3 channels, on the other hand, require depolarized potentials near −45 mV or higher to open and therefore are not expected to be conducting currents at hyperpolarized potentials. Thus, both Kir2.1 and Kv1.3 contribute to the membrane potential in microglia—Kir2.1 by keeping the membrane closer to the K^+^ reversal potential and Kv1.3 by preventing the potential to rise above its threshold potential.

Interestingly, RMP in undifferentiated rat microglia has been reported to fluctuate between a −35 mV peak and a −70 mV peak, with the former shifted to −45 mV by LPS (Boucsein et al., 2003; Chung et al., 1999; Norenberg, Gebicke‐Haerter, & Illes, 1994; Visentin, Agresti, Patrizio, & Levi, 1995). We suspect that these observed discrepancies in RMP are attributable to a species‐specific channel expression response since Kir2.1 expression has been reported to be drastically suppressed in mouse microglia but retained in rat microglia after LPS stimulation (Chung et al., 1999; Draheim et al., 1999; Nguyen et al., 2017; Norenberg, Gebicke‐Haerter, & Illes, 1992, 1994).

In addition to its contribution to maintaining a negative RMP, perhaps the most understudied, but equally important role of Kv1.3 is to offset extreme potential changes during active membrane depolarization events. Both Kv1.3‐expressing CHO cells and microglia are completely resistant to depolarizations elicited by current injections. Furthermore, Kv1.3 activity in microglia similarly counteracts evoked depolarizations triggered by puffs of 100 μM ATP, which activate P2X4 receptors and lead to fast depolarizing cationic currents and massive depolarization as shown in this report and others (Visentin et al., 1999). Interestingly, this ability of Kv1.3 to tune depolarizing events has been alluded to in previous studies showing that non‐proliferating Kv1.3‐expressing microglia have shorter ATP‐evoked depolarizations than proliferating Kv1.3‐negative, Kir2.1‐positive cells despite possessing similar ATP‐induced inward currents (Kettenmann, Banati, & Walz, 1993; Norenberg, Langosch, Gebicke‐Haerter, & Illes, 1994). Functionally, Kv1.3 channels in microglia not only influence the basal driving force for Ca^2+^ entry during rest but also limit calcium overloading and steep membrane potential changes during active Ca^2+^ influx that might otherwise be detrimental to overall microglial health.

We demonstrate that the ability of Kv1.3 to normalize evoked membrane depolarization can effectively be limited by the Kv1.3 blocker ShK‐223. Using a combination of immunohistofluorescence staining and electrophysiological recordings we also showed that P2X4 is upregulated in activated microglia isolated from the brains of transgenic CX3CR1^+/GFP^ mice following induction of ischemic stroke, in line with previous findings showing an upregulation of P2X4 in ischemic conditions (Cavaliere et al., 2003; Li et al., 2011; Wixey, Reinebrant, Carty, & Buller, 2009). This change is concurrent with the appearance of Kv1.3, which we and others have consistently shown to be upregulated in activated microglia in both rodent and human stroke brains (Chen et al., 2016; Chen et al., 2018; Gao et al., 2019). Taken together, these findings demonstrate the relevance of Kv1.3 in purinergic calcium signaling since the depolarization associated with ATP‐induced entry of Ca^2+^ and other cations is strong enough to open Kv1.3 channels. Indeed, blocking Kv1.3 with PAP‐1 reduced microglia activation and overall brain levels of the inflammatory cytokines IL‐1β and IFN‐γ, leading to smaller infarct areas and improved neurological deficit scores in a mouse model of ischemic stroke (Chen et al., 2018). PAP‐1 also reduced NF‐κB activation, IL‐1β and TNF‐α expression, NO generation, and p38MAPK phosphorylation associated with AβO‐induced microglial activation (Maezawa et al., 2018). These affected pro‐inflammatory responses and cytokines are also associated with the neurotoxic functions of P2X4 in microglia (Li et al., 2011; Verma et al., 2017). Thus, blocking Kv1.3 prevents normalization of P2X4‐mediated depolarization and disrupts Ca^2+^ influx leading to reduced microglia activation.

P2X4 expression in both LPS‐differentiated cultured microglia and acutely isolated microglia from brains of animals 24 hr after ICV injection of LPS was significantly reduced, contrasting findings from our MCAO ischemic stroke microglia and a study using the mouse BV2 microglial cell line (Raouf, Chabot‐Dore, Ase, Blais, & Seguela, 2007). Since microglia in general are known to display high phenotypic heterogeneity (Horvath, Nutile‐McMenemy, Alkaitis, & Deleo, 2008; Lawson, Perry, Dri, & Gordon, 1990; Ren, Lubrich, Biber, & Gebicke‐Haerter, 1999), this discrepancy suggests that acute LPS‐treated microglia might have acquired an activation state distinct from BV2 cells or microglia in the ischemic brain, which are exposed to multiple stimuli including hypoxia, ATP, glutamate and neuronal debris. The PAMP molecule LPS activates microglia mainly through the toll‐like receptor 4 (Chakravarty & Herkenham, 2005; Doyle & O'Neill, 2006), while the DAMP molecule ATP activates purinergic receptors. Interestingly, our findings are in line with studies reporting a general downregulation of ATP‐gated current responses found in mouse primary microglia following treatment with LPS (Hoffmann, Kann, Ohlemeyer, Hanisch, & Kettenmann, 2003; Moller, Kann, Verkhratsky, & Kettenmann, 2000). Functionally, it could be postulated that undifferentiated surveillant microglia are more attuned to changes in their microenvironment because they need to respond immediately to distress signals released early on during brain insults. In contrast, activated microglia adopting an effector phenotype possess an increased energy and calcium demand. A persistent elevation of basal [Ca^2+^]i occurred in parallel with the suppression of evoked calcium signaling in LPS‐differentiated microglia (Hoffmann et al., 2003) suggesting increased cytoplasmic Ca^2+^ as the basis for microglia effector functions. Therefore, it is highly plausible that the consistent upregulation of Kv1.3 in LPS‐treated cells is associated with providing sustaining repolarizing currents for this basal Ca^2+^ increase as well as optimizing Ca^2+^ entry through reduced P2X4 receptors.

We elected not to investigate the role of Kv1.3 in relation to P2X7 because we could detect only an insignificant amount of P2X7 currents in our in vitro microglia. Additionally, LPS differentiation, both in vitro and in vivo, induced a significant downregulation of P2X7 expression. Although this low affinity P2X receptor is known to be associated with pro‐inflammatory microglia functions such as IL‐1β release (Di Virgilio, Ceruti, Bramanti, & Abbracchio, 2009) and is upregulated in microglia under ischemic conditions (Eyo, Miner, Ahlers, Wu, & Dailey, 2013; Melani et al., 2005), its actual contribution to ischemic damage is not clear. Genetic deletion of P2X7 receptors and treatment with P2X7 antagonists did not affect ischemic or excitotoxic cell death, suggesting that P2X7 receptors are not primary mediators of experimentally induced neuronal death thus questioning the therapeutic potential of reducing P2X7 activity (le Feuvre, Brough, Touzani, & Rothwell, 2003). Furthermore, activation of P2X7 receptors by high ATP concentrations leads to the formation of both an ion channel pore and a macroscopic pore. While Kv1.3 inhibition might be able to counter Ca^2+^ flux through the P2X7 ion channel pore, it might not be suitable or effective in modifying the activity of the macroscopic pore, which is permeable to hydrophilic moieties of up to 900 Da (Surprenant, Rassendren, Kawashima, North, & Buell, 1996). This was suggested in a study describing that neither MgTX, a widely used peptidic Kv1.3 blocker, nor high potassium affect IL‐1β release triggered by BzATP in experiments with human macrophages primed with LPS (Mackenzie, Chirakkal, & North, 2003). On the contrary, evidence exists questioning the pro‐inflammatory function of P2X7 in microglia and showing that the P2X7 pore can facilitate trophic function in driving microglia into a proliferative state (Monif, Reid, Powell, Smart, & Williams, 2009).

We also did not investigate the role of Kv1.3 in regulating other microglial calcium entry pathways induced by low levels of ATP in this study. At 0.1 mM, ATP can not only activate P2X4 receptors but also trigger a P2Y purinoceptor‐dependent depletion of internal stores (McLarnon, 2005). Thus, we cannot rule out the possibility that the micromolar ATP used in this study might elicit additional store‐operated Ca^2+^ entry mediated by CRAC channels. Given that Kv1.3 inhibition effectively reduces CRAC‐mediated Ca^2+^ influx in T‐cells (Cahalan & Chandy, 2009), it is possible that a reduction of CRAC contributes to the immunomodulatory action of Kv1.3 inhibition in microglia. Inhibiting CRAC channels with CM‐EX‐137 has been shown to decrease lesion size, brain hemorrhage, and improve neurological deficits while decreasing microglial activation, iNOS, Orai1, and STIM1 levels in an animal model of traumatic brain injury (Mizuma et al., 2019). Of note, CRAC channels have been linked to the calcium‐activated potassium channel KCa3.1, another potassium channel found to be upregulated in activated microglia. Our previous work demonstrated that both KCa3.1 deletion and pharmacological inhibition reduce infarction and associated inflammatory responses in rodent models of ischemic stroke (Chen et al., 2016; Chen, Raman, Bodendiek, O'Donnell, & Wulff, 2011). KCa3.1 has not been shown to couple to P2X4 in microglia but it has been shown to associate with the P2Y2 receptor. In ovarian cancer cells activation and opening of KCa3.1 channels induced by intracellular calcium release from internal stores caused by activation of P2Y2 receptors, significantly influences cellular motility (Robles‐Martinez et al., 2017). In microglia, this P2Y2‐KCa3.1 axis also regulates migration—following P2Y receptor activation by UTP, calcium influx through CRAC/Orai1 channels replenishes the depleted stores, and this is facilitated by KCa3.1 channel activity (Ferreira & Schlichter, 2013). UTP‐stimulation of P2Y2 receptors have also been shown to activate KCa3.1 channels in Xenopus oocytes (Hede, Amstrup, Klaerke, & Novak, 2005) and macrophages (Gao, Hanley, Rinne, Zuzarte, & Daut, 2010). Thus, it is plausible that KCa3.1 activity could also be functionally coupled to P2X4 receptor activity.

In summary, we combined whole‐cell voltage‐clamp electrophysiology and qPCR to show a differential expression pattern for Kv1.3 and P2X4 in LPS‐differentiated microglia and microglia from ischemic stroke brains. Interestingly, we could confirm the previously reported gender differences in microglial P2X4 expression (Mapplebeck et al., 2016) in vivo following ischemic stroke, but not in neonatal microglia cultures. Kv1.3 expression in contrast exhibited no gender differences in microglia in vitro or in vivo. The presence of Kv1.3 channels contributes not only to maintenance of the RMP but also regulates active potential changes downstream of calcium influx through P2X4 receptors. Inhibiting Kv1.3 channels with ShK‐223 disrupts the driving force for calcium entry and completely nullified the ability of Kv1.3 to prevent excessive depolarizations leading to reduced calcium transients through P2X4 receptors. Our report thus links Kv1.3 function to P2X4 activity as the underlying mechanism by which targeted Kv1.3 blockade reduces microglia‐mediated neuroinflammation.

## Supporting information


**Supplemental Figure S1** Acute inhibition of Kv1.3 reduces ATP‐mediated Ca^2+^ entry. Application of the small molecule inhibitor PAP‐1 (100 nM) for 5 minutes after the first Ca^2+^ transient reduced the amplitude of the second Ca^2+^ transient in LPS‐treated microglia (b) but not in untreated (a) or IL‐4 treated microglia (c). 0.1 mM ATP was used for induction of Ca^2+^ transients. Undifferentiated microglia without PAP‐1 (second/first peak ratio of 0.66 ± 0.10; n = 502 cells from N = 8 experiments) and with PAP‐1 (second/first peak ratio of 0.64 ± 0.12; *p* = 0.6837; n = 377 cells from N = 6 experiments). LPS (300 ng/ml) treated microglia without PAP‐1 (second/first peak ratio of 0.50 ± 0.09; n = 280 cells from N = 6 experiments) and with PAP‐1 (second/first peak ratio of 0.26 ± 0.13; *p* = 0.0059; n = 213 cells from N = 5 experiments). IL‐4 (20 ng/ml) treated microglia without PAP‐1 (second/first peak ratio of 0.56 ± 0.12; n = 249 cells from N = 3 experiments) and with PAP‐1 (second/first peak ratio of 0.70 ± 0.05; *p* = 0.2220); n = 179 cells from N = 3 experiments). Statistical analysis applied was a paired t‐Test. Error bars indicate mean ± *SD*.
**Figure S2**. Validation of secondary antibody. Sample immunofluorescence staining of 14‐μM thick coronal brain sections from the 6‐mm depth showing no immunoreactivity when the Alexa Fluor®647 conjugated secondary antibody (1:1000, Life Technologies) is used alone without primary antibodies in either the contralateral or ipsilateral side of the Cx3CR1^+/EGFP^ (green) MCAO brain. Each channel was analyzed on n = 3–4 coronal sections. Scale bar indicates 100 μm.
**Figure S3.** Validation of polyclonal rabbit anti‐P2X4 antibody. Sample immunofluorescence staining of 14‐μM thick Cx3CR1^+/EGFP^ (green) MCAO brain coronal brain sections from the 6‐mm depth showing no immunoreactivity with the rabbit anti‐P2X4 antibody (Alomone, APR‐002, 1:1000) in the ipsilateral brain after preincubation with blocking peptide for 45 minutes at RT in contrast to clear staining without the blocking peptide. Each channel was analyzed on n = 3–4 coronal sections. Scale bar indicates 100 μm.
**Figure S4.** Validation of polyclonal rabbit anti‐Kv1.3 antibody. Sample immunofluorescence staining of 14‐μM thick Cx3CR1^+/EGFP^ (green) MCAO brain coronal brain sections from the 6‐mm depth showing no immunoreactivity with the rabbit a polyclonal rabbit anti‐Kv1.3 antibody (Alomone, APC‐101, 1:1000) in the ipsilateral brain after preincubation with the blocking peptide for 45 minutes at RT in contrast to clear staining without the blocking peptide. Each channel was analyzed on n = 3–4 coronal sections. Scale bar indicates 100 μm.

NguyenSupplementFigs

## Data Availability

The data that support the findings of this study are available from the corresponding author upon reasonable request.
