# Supplementary figures and images for: Biophysical basis for Kv1.3 regulation of membrane potential changes induced by P2X4‐mediated calcium entry in microglia
Source: Glia. 2020 Jun 11;68(11):2377–94. doi: 10.1002/glia.23847 (PMC7540709; doi:10.1002/glia.23847)

Figure S1.

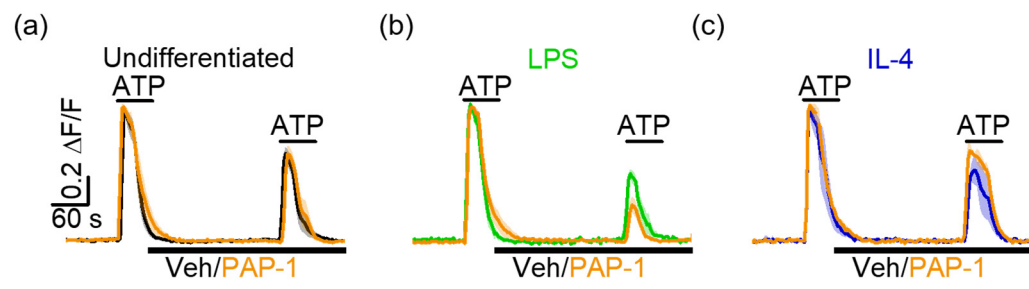

Figure S2.

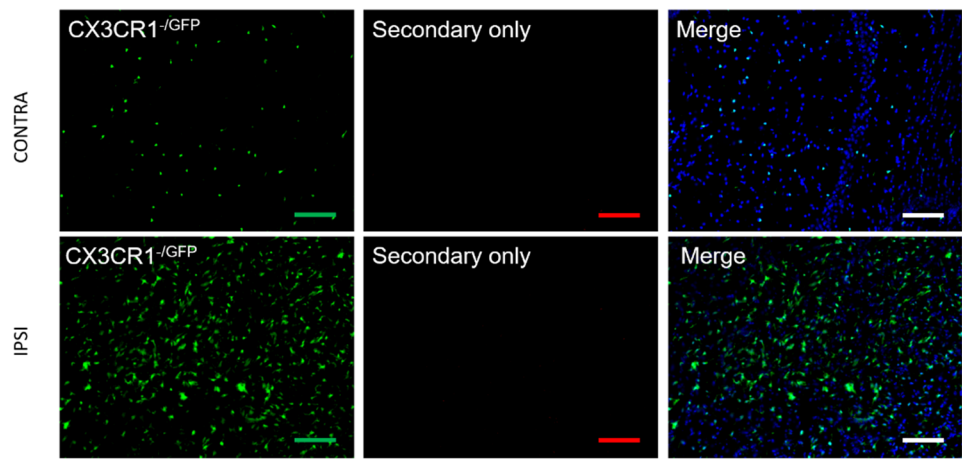

Figure S3.

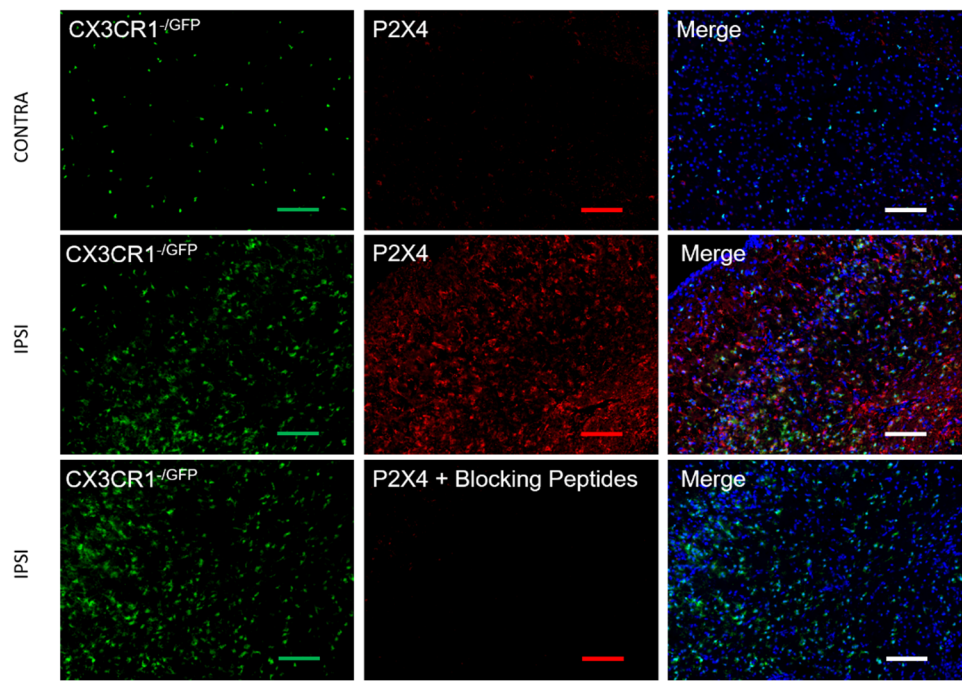

Figure S4.

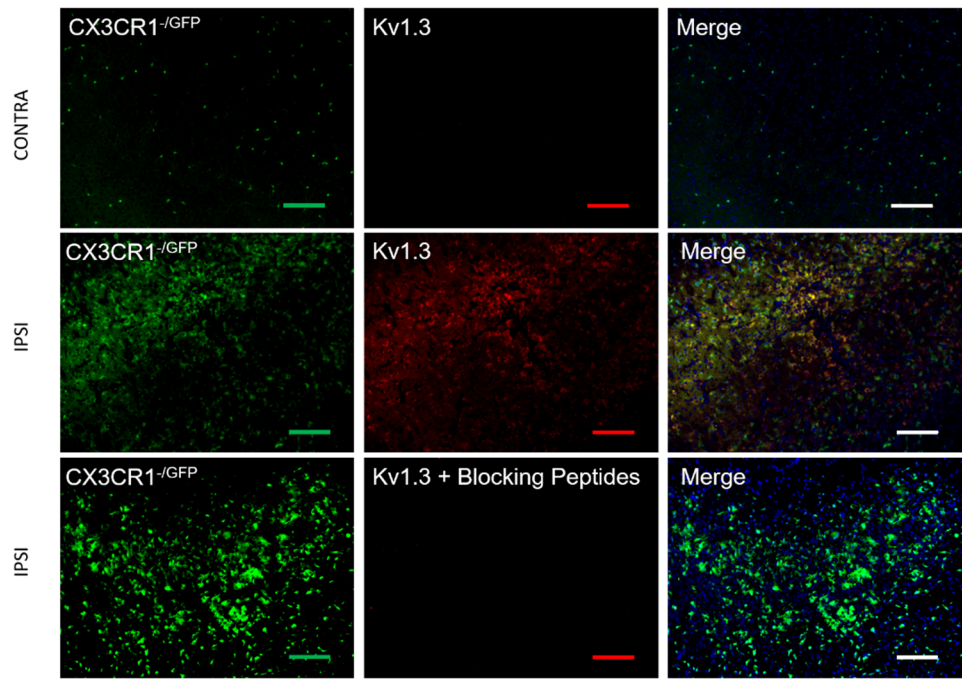

Supplement: Supplementary file 2 — NguyenSupplementFigs [file GLIA-68-2377-s002.pdf]
