# Supplementary material for: Biophysical basis for Kv1.3 regulation of membrane potential changes induced by P2X4‐mediated calcium entry in microglia
Source: Glia. 2020 Jun 11;68(11):2377–94. doi: 10.1002/glia.23847 (PMC7540709; doi:10.1002/glia.23847)
Supplement: Supplementary file 1 — Supplemental Figure S1 Acute inhibition of Kv1.3 reduces ATP‐mediated Ca2+ entry. Application of the small molecule inhibitor PAP‐1 (100 nM) for 5 minutes after the first Ca2+ transient reduced the amplitude of the second Ca2+ transient in LPS‐treated microglia (b) but not in untreated (a) or IL‐4 treated microglia (c). 0.1 mM ATP was used for induction of Ca2+ transients. Undifferentiated microglia without PAP‐1 (second/first peak ratio of 0.66 ± 0.10; n = 502 cells from N = 8 experiments) and with PAP‐1 (second/first peak ratio of 0.64 ± 0.12; p = 0.6837; n = 377 cells from N = 6 experiments). LPS (300 ng/ml) treated microglia without PAP‐1 (second/first peak ratio of 0.50 ± 0.09; n = 280 cells from N = 6 experiments) and with PAP‐1 (second/first peak ratio of 0.26 ± 0.13; p = 0.0059; n = 213 cells from N = 5 experiments). IL‐4 (20 ng/ml) treated microglia without PAP‐1 (second/first peak ratio of 0.56 ± 0.12; n = 249 cells from N = 3 experiments) and with PAP‐1 (second/first peak ratio of 0.70 ± 0.05; p = 0.2220); n = 179 cells from N = 3 experiments). Statistical analysis applied was a paired t‐Test. Error bars indicate mean ± SD. Figure S2. Validation of secondary antibody. Sample immunofluorescence staining of 14‐μM thick coronal brain sections from the 6‐mm depth showing no immunoreactivity when the Alexa Fluor®647 conjugated secondary antibody (1:1000, Life Technologies) is used alone without primary antibodies in either the contralateral or ipsilateral side of the Cx3CR1+/EGFP (green) MCAO brain. Each channel was analyzed on n = 3–4 coronal sections. Scale bar indicates 100 μm. Figure S3. Validation of polyclonal rabbit anti‐P2X4 antibody. Sample immunofluorescence staining of 14‐μM thick Cx3CR1+/EGFP (green) MCAO brain coronal brain sections from the 6‐mm depth showing no immunoreactivity with the rabbit anti‐P2X4 antibody (Alomone, APR‐002, 1:1000) in the ipsilateral brain after preincubation with blocking peptide for 45 minutes at RT in contrast to clear [file GLIA-68-2377-s001.doc]

UNIVERSITY OF CALIFORNIA DAVIS

BERKELEY • DAVIS • IRVINE • LOS ANGELES • MERCED • RIVERSIDE • SAN DIEGO • SAN FRANCISCO SANTA BARBARA • SANTA CRUZ

DEPARTMENT OF PHARMACOLOGY

SCHOOL of MEDICINE

ONE SHIELDS AVENUE

UNIVERSITY OF CALIFORNIA, DAVIS

DAVIS, CALIFORNIA 95616-8636

Phone: 530-752-3200

Fax: 530-752-7710

April 16^th^, 20202

**Re:** Resubmission of GLIA-00427-2019

Dear Dr. Ransom,

We would like to resubmit our manuscript entitled *"* *Biophysical Basis for Kv1.3 Regulation of Membrane Potential Changes Induced by P2X4 mediated Calcium Entry in Microglia” Activated Microglia"* to *Glia*.

We believe that we have addressed all concerns raised by the two reviewers in a meaningful way by either performing additional experiments and/or doing better statistics. We apologize that it took us three months to make these changes because we had to prepare several neonatal microglia cultures and would like to thank you again for allowing us the necessary time. A detailed summary of the changes and our responses to the reviewer comments is enclosed. We have also carefully proof-read the manuscript again in order to minimize typographical, grammatical, and bibliographical errors. We hope that the manuscript is now acceptable for publication in *Glia*.

Sincerely,

Heike Wulff, Ph.D.

Professor, Department of Pharmacology

University of California, Davis

Genome and Biomedical Sciences Facility, Room 3502

451 Health Sciences Drive

Davis, CA 95616

Phone: 530 754-6135

Fax: 530 752 7710

Email: [hwulff@ucdavis.edu](mailto:hwulff@ucdavis.edu)

*We would like to thank both reviewers for their insightful comments which have significantly improved our manuscript.*

**Reviewer:1

Comments to the Author**

This is an interesting paper that provides a role for Kv1.3 channels in the pro-inflammatory functions of microglia via maintaining the membrane potential necessary for Ca2+ influx through P2X4 receptors. The information provided are important in the context of neuroinflammation that plays an important role in neurodegenerative diseases and stroke. The experiments are well designed and executed, and the appropriate controls are in place. However, there are concerns that reduce my enthusiasm.

1.      It is not clear how the statistical analyses have been done. It is reported in the Method section that the data have been analyzed using a paired Student's t-test, however this may not be the appropriate test for all the comparisons.  If other tests have been used, they should be reported in the Method Section and in the legends of figures and tables. The results are difficult to interpret if the statistical analysis is not the correct one. Was a two-way ANOVA used for panels e and h of Figure 5? Also, Table 2: are you sure that there is a significant difference with a p<0.001 between the RMP of unstimulated cells without and with ShK-223 (-88.90+/-5.20 vs -80.90+/-11.73 mV, respectively)?  In the legend of Table 2 it is reported that “paired t-test was used for direct comparison between two conditions” however it is not clear what the “two conditions” are. A paired t-test should be applied when before and after values in a single cell or tissue are compared and it requires equal sample size. Same considerations apply for Table 2 where all the comparisons appeared to have been done using paired t-test. The authors may want to seek the assistance of a statistician.

*Response: We would like to thank the reviewer for this comment. After consultation with Dr. Daniel J. Tancredi, PhD, who is an Associate Professor in Residence in the Department of Pediatrics at UC Davis and the Director of the Statistics and Data Management Core on a program project that my laboratory is part of, we have now updated our statistical analysis and clarified which test was used for each individual panel of each figure and table by indicating this in the legends. Thank you. This has improved our study.*

*We are now using one-way ANOVA followed by post-hoc Tukey-Cramer’s method to determine statistical significance in every figure where we compare three groups or more. Comparison of measurements made within the same group of cells before and after treatment such as ShK-223 or ivermectin are made with a paired t-Test. All comparisons made between two groups of cells subjected to different stimulation conditions (e.g. untreated versus LPS stimulated) are analyzed by unpaired t-Test.*

2.      The possible role of another K+ channels expressed in microglia, KCa3.1, in limiting P2X4-mediated Ca2+ influx has not been considered (neither experimentally tested nor discussed). The authors and other groups have shown that KCa3.1 channels are involved in microglia activation.

*Response: The reviewer is of course correct in pointing out that KCa3.1 could also play a role in limiting P2X4-mediated calcium influx. Potassium currents in general repolarize membrane potential, and currents carried by KCa3.1 are expected to contribute a repolarizing force in microglia. Our previous work with the KCa3.1 inhibitor TRAM-34 in a stroke model demonstrates an important role for KCa3.1 in microglia activation* ***(Chen, Yi-Je, et al. "The KCa3. 1 blocker TRAM-34 reduces infarction and neurological deficit in a rat model of ischemia/reperfusion stroke." Journal of Cerebral Blood Flow & Metabolism 31.12 (2011): 2363-2374)****. However, it is currently unknown whether KCa3.1 in microglia is functionally coupled to P2X4. Studies on the purinergic P2Y2 receptor hint at a cross talk between KCa3.1 and at least GPCR-type purinergic receptors. For example, motility of ovarian cancer SKOV-3 cells and neoplastic cells from human ovarian tumor biopsies is regulated by the activation and opening of KCa3.1 channels, which is tied to ATP-induced intracellular calcium release from internal stores caused by activation of P2Y2 receptors* ***(Robles-Martínez, L., et al. "KCa3.1 activation via P2Y2 purinergic receptors promotes human ovarian cancer cell (Skov-3) migration." Scientific reports 7.1 (2017): 1-14.)*** *In microglia, this P2Y2-KCa3.1 axis also regulates migration - following P2Y receptor activation by UTP, calcium influx through CRAC/Orai1 channels replenishes the depleted stores, and this is facilitated by KCa3.1 channel activity (****Ferreira, Roger, and Lyanne C. Schlichter. "Selective activation of KCa3. 1 and CRAC channels by P2Y2 receptors promotes Ca2+ signaling, store refilling and migration of rat microglial cells." PLoS One 8.4 (2013)****. UTP-stimulation of P2Y2 receptors has also been shown to activate KCa3.1 channels in Xenopus oocytes (****Hede SE, Amstrup J, Klaerke DA, Novak I (2005) P2Y2 and P2Y4 receptors regulate pancreatic Ca2+-activated K+ channels differently. Pflugers Arch 450: 429–436****) and macrophages (****Gao YD, Hanley PJ, Rinne S, Zuzarte M, Daut J (2010) Calcium-activated K+ channel (KCa3.1) activity during Ca2+ store depletion and store-operated Ca2+ entry in human macrophages. Cell Calcium 48: 19–27****). Thus, we agree with the reviewer that it is certainly possible that KCa3.1 activity is functionally coupled to P2X4 receptor activity. Interestingly, our preliminary results show a lack of effect of 1 uM TRAM-34 (selective KCa3.1 blocker) on ATP-triggered calcium signaling measured by Fluo-4 AM in primary microglia under differentiation conditions that were previously shown to induced KCa3.1 expression, namely differentiation with ATP or IL-4 for 24 hours.*

KCa3.1 channel inhibition with TRAM-34 (1uM) did not alter the Fluo-4 AM calcium indicator fluorescence signal elicited by 0.1 mM ATP in microglia differentiated with (a) ATP (500 uM, 24h) and (b) IL-4 (20ng/mL, 24h). Change in [Ca^2+^]_i_ reported as ΔF (change in fluorescence after baseline‑subtraction). Scale bars indicate 10 arbitrary unit (A.U.). Measurements from a total of 125-189 cells from at least three independent experiments.

*This is of course very preliminary data and we believe that a possible functional role of KCa3.1 in P2X4-mediated calcium influx in microglia necessitates a thorough investigation separate from the current paper focused on Kv1.3. However, we now include a short paragraph in the Discussion section about the possible importance of KCa3.1 in microglia function. At this point we believe that there might be a “division of labor” between Kv1.3 and KCa3.1 in microglia and that Kv1.3 regulates P2X4-mediated calcium influx, while KCa3.1 might be more important for calcium signaling events involving stores and P2Y receptors. But this would of course require further studies.*

3.      Line 437: Shouldn’t it read 21 mV instead of 11 mV? Based on Table 2, ShK-223 increases the RMP of LPS-treated cells from -66 mV to -45 mV. All this section (starting at line 429) is not very clear and would benefit from careful re-writing as it is very important to support the conclusion that Kv1.3 confers to LPS-activated microglia the ability to resist AID.

*Response: We thank the reviewer for identifying this typographical error. This has now been corrected to “21 mV” in the corresponding sentence. We also have revised our current clamp data in the original Table 2 by splitting it into two separate tables - data recorded from only cells subjected to inhibition by ShK-223 (Table 3) and data from the rest of the cells recorded that were not treated with ShK-223 (Table 2). We have accordingly revised our analysis and carefully re-wrote this section.*

4.      Line 463: The data presented do not support the conclusion that “Similarly, stimulation of microglia with IL-4 also led to reduced Ca2+ flux reflecting the downward trend in current density and reduced expression (Figures 2 and 3)”. While Fig. 3 shows a significant reduction in P2X4 mRNA by IL-4, figure 2g shows that IL-4 has no significant effect on P2X4 current. This latter finding makes very unlikely that these channels play a role in reduced Ca2+ fluxes in IL4-stimulated microglia. The authors should consider alternative explanations.

*Response: The fact that IL-4 differentiated cells show a change in the current density for P2X4 on the membrane but reduced ATP-induced calcium signaling is likely due to the lack of restorative current for the driving force for calcium. We now have revised our explanation in line 463 (now line 445) to: “Interestingly, stimulation of microglia with IL-4 lowered Ca^2+^ entry upon stimulation with ATP compared to undifferentiated cells (Figure 6c) but less than LPS stimulation did. Since IL-4 did not reduce P2X4 current density (Figure 2g), and* *inhibiting Kv1.3 currents had a major impact on membrane potential (Figure 5), this reduction in Ca^2+^ flux is likely due to the lack of a restoration of the driving force for calcium entry due to the very low levels of Kv1.3 channels in IL-4 differentiated cells”.*

5.      Line 498: How cell capacitance was measured should be reported in the method section.

*Response: We apologize for omitting this information. Cell capacitance was directly measured by a lock-in function built in the HEKA PatchMaster 9 data acquisition software. This has been clarified in the methods section.*

6.      Fig. 6: Why were different concentrations of ATP used (0.1 mM in panel a, and 30 uM in panel b)? Do these concentrations produce a similar effect?  What concentrations of ATP where used for the experiments in panel c?

*Response: We apologize for this confusion, which arose from an error made when copying and pasting the legend of Figure 2 when preparing the legend for Figure 6. The concentrations of ATP used in studying the effect of ivermectin in our electrophysiology experiments (Figure 2) and calcium imaging experiments (Figure 6) are different. Since the EC_50_ of ATP for activating mouse P2X4 receptors is approximately 2.3 µM (****Jones et al., 2000 Br J Pharmacol. 2000 Jan; 129(2): 388–394****), concentrations of 30 microM and 100 microM will activate a robust response that is exclusively mediated by P2X4 and not P2X7, which has higher sensitivity to BzATP. We used 100 microM ATP in all figures, including Figure 6, in our report to elicit maximal P2X4-mediated calcium responses. Figure 2b, the ONLY exception in our report, on the other hand, is intended to further confirm the presence of P2X4 using its sensitivity to ivermectin, a selective positive modulator of P2X4. Hence, we used 30 µM ATP in Figure 2b to increase the dynamic range of ivermectin’s potentiating effect on P2X4 currents measured by electrophysiology. We have now corrected this error and edited our Figure 6 legend.*

Also, panels f and g are not mentioned in the figure legend.

*Response: We apologize for this typographical error. All panels are now mentioned in the legend of Figure 6.*

The Y axis in the Ca2+ traces is not clear. What does 1ΔF and 20%ΔF stand for?  This figure legend should be re-written.

*Response: We measured fluorescence changes indicated by the Fluo-4 AM dye as fold change over baseline after background subtraction (ΔF/F). In panels 6c, we normalized these fluorescence changes measured in LPS and IL-4 cells to that of undifferentiated cells. Similarly, in panels 6d to 6f, we normalized fluorescence changes after ShK-223 treatment to changes measured before ShK-223. In Figures 6a and 6b, to compare the effect of BzATP or ATP in the presence of ivermectin to the effect of ATP alone, fluorescence changes were measured as area under the curve (AUC) and normalized to the AUC of ATP. Scale bars are set to 0.2 (or 20%) of maximal normalized fluorescence (1ΔF/F). This is now explained in the legend.*

7.      The legend for panel a of Fig. 6 reports n=2 for BzATP while there are 3 points in the bar graph. Also the concentration of BzATP is missing.

*Response: We apologize for this typographical error. It has been corrected. Thank you for finding it.*

8.      Fig. 7. Since polyclonal antibodies have been used, their specificity should be shown and unstained controls should also be reported as Supplemental data.

*Response: We agree with the reviewer’s concern regarding the use of polyclonal antibodies in our immunohistochemical data. We now have included new supplemental Figures (Figures S2-S4) validating the specificity of our antibodies. We demonstrate that the staining of the primary antibodies can be prevented by using blocking peptides and that the secondary antibodies do not produce any unspecific staining.*

9.      Line 331: it should be reported that BzATP is a P2X7 receptor agonist.

*Response: The sentence in line 331 has been edited to “Based on their differential sensitivity to nucleotide derivatives (Chessell et al., 1998; Townsend-Nicholson et al., 1999), P2X4 and P2X7 currents* *were measured as inward currents elicited by saturating concentrations of their selective agonist, ATP for P2X4 and BzATP for P2X7, respectively.*

10.     Line: 458:  “more than 3-fold” should read “3-fold”

*Response: Thank you. This has been corrected.*

**Reviewer:2**
 **Comments to the Author**

Nguyen et al. analyzed the molecular mechanisms underlying the therapeutic potential of Kv1.3 inhibition in microglia for preventing neuroinflammation. Nguyen et al. showed by whole-cell voltage-clamp electrophysiology and qPCR a stimulus-dependent differential expression pattern for Kv1.3 and P2X4 in cultured microglia. The presence of Kv1.3 channels rendered microglia more resistant to depolarization produced by ATP-mediated P2X4 receptor activation, while inhibiting Kv1.3 channels with ShK-223 completely neutralized the ability of Kv1.3 to normalize the membrane potential changes.

Data are novel and link the Kv1.3 function to P2X4 receptor mediated signaling as one of the underlying mechanisms. Expression of the voltage-gated potassium channel Kv1.3 is required for microglia activation and pharmacological inhibition of Kv1.3 are effective in reducing microglia activation and the associated inflammatory responses.

Results are well presented and outcomes are carefully discussed.

However, there some points to be completed to justify the claims made:
1.      It is claimed in the abstract that ‘Kv1.3 function is linked to P2X4 receptor mediated signaling as one of the underlying mechanisms by which Kv1.3 blockade reduces microglia-mediated inflammation’. However, no data on reduced microglia-mediated inflammation after blockade of Kv1.3 are shown. How does the inflammatory gene transcription profile of figure 3b change after additionally inhibiting Kv1.3 channels with ShK-223?

*Response: We would like to thank the reviewer for this comment. We have previously reported the effectiveness of blocking Kv1.3 in reducing microglia-mediated inflammation in multiple models. Using primary mouse microglia in vitro, we showed that both ShK-186 and PAP-1 significantly reduce cytokine expression and production in microglia stimulated with LPS* ***(Nguyen, Hai M., et al. "Differential Kv1. 3, KCa3. 1, and Kir2. 1 expression in “classically” and “alternatively” activated microglia." Glia 65.1 (2017): 106-121).*** *In a model of ischemic stroke* ***(Chen, Yi‐Je, et al. "Inhibition of the potassium channel Kv1. 3 reduces infarction and inflammation in ischemic stroke." Annals of clinical and translational neurology 5.2 (2018): 147-161),*** *we showed that blocking Kv1.3 with the small molecule PAP-1 reduces microglia activation in hippocampal slices and dose-dependently reduced infarct area as well as brain levels of IL-1β and IFN-γ without affecting IL-10 and brain-derived nerve growth factor (BDNF) levels in mouse MCAO brains. We also showed that PAP-1 reduces primary microglia activation induced by amyloid beta oligomers and the associated IL1-beta, TNF-alpha and iNOS expression and significantly improved behavioral deficits in the 5xFAD model mouse model of Alzheimer’s disease* ***(Maezawa, Izumi, et al. "Kv1. 3 inhibition as a potential microglia-targeted therapy for Alzheimer’s disease: preclinical proof of concept." Brain 141.2 (2017): 596-612).*** *These findings were the rationale and motivation for this current report, in which we focus mainly on providing insights into the mechanism of action of Kv1.3 inhibition that yielded the positive outcomes mentioned in the papers mentioned.*

*However, in order to address the reviewer’s comment, we have now also added data showing that ShK-223, the ShK derivative used in this study, changes the inflammatory gene transcription profile in microglia (see next comment).*

2.      Likewise, it would be important to understand whether blockade of the Kv1.3 channels with ShK-223 is changing the gene transcription and/or expression of the P2X4 receptor.

*Response: We agree with the reviewer’s comment about the importance of determining the effect of Kv1.3 inhibition on the expression of P2X4 receptors. The time afforded during this revision has allowed us to examine the effects of ShK-223 on the microglial membrane protein and mRNA expression of not just P2X4, but also P2X7, Kv1.3, and Kir2.1 in three independent microglia cultures from three litters. This new data is now presented as a new figure (Figure 8) in our revised manuscript. In brief, while blocking Kv1.3 with ShK-223 did prevent the LPS-induced decrease and increase in P2X4 and Kv1.3 membrane current density, respectively (Figure 8c), it did not affect the increase in cell size as measured in pF or prevent the suppression of Kir2.1 current density. These changes were paralleled by changes in mRNA expression of these channels (Figure 8a). Interestingly, ShK-223 also prevented the reduction of P2X7 current expression but had no effect on P2X7 mRNA, suggesting that Kv1.3 is not involved in the transcriptional regulation of this receptor. Overall, our data suggest that Kv1.3 channel function is required for the polarization of activated microglia into a specific subset but not for microglia activation per se since it did not prevent the increase in cell size. Based on the finding that ShK-233 significantly reduced TNF-α mRNA and induced a downward trend in IL-1β and iNOS mRNA expression at 24 h (Figure 8b), we conclude that Kv1.3 is specifically required for the polarization of activate microglia into a pro-inflammatory state. These results are in line with our previously published work showing that Kv1.3 inhibition is beneficial in alleviating proinflammatory microglia responses (Nguyen et al., 2017; Chen et al, 2018; Maezawa et al., 2018).*
